# Patterns of gene recombination shape *var *gene repertoires in *Plasmodium falciparum: *comparisons of geographically diverse isolates

**DOI:** 10.1186/1471-2164-8-45

**Published:** 2007-02-07

**Authors:** Susan M Kraemer, Sue A Kyes, Gautam Aggarwal, Amy L Springer, Siri O Nelson, Zoe Christodoulou, Leia M Smith, Wendy Wang, Emily Levin, Christopher I Newbold, Peter J Myler, Joseph D Smith

**Affiliations:** 1 Seattle Biomedical Research Institute, 307 Westlake Avenue North, Suite 500, Seattle, WA 98109-5219, USA; 2 Molecular Parasitology Group, Weatherall Institute of Molecular Medicine, University of Oxford, John Radcliffe Hospital, Oxford OX3 9DS, UK; 3 Department of Pathobiology, University of Washington, Seattle, WA 98195, USA

## Abstract

**Background:**

*Var *genes encode a family of virulence factors known as PfEMP1 (*Plasmodium falciparum *erythrocyte membrane protein 1) which are responsible for both antigenic variation and cytoadherence of infected erythrocytes. Although these molecules play a central role in malaria pathogenesis, the mechanisms generating variant antigen diversification are poorly understood. To investigate *var *gene evolution, we compared the variant antigen repertoires from three geographically diverse parasite isolates: the 3D7 genome reference isolate; the recently sequenced HB3 isolate; and the IT4/25/5 (IT4) parasite isolate which retains the capacity to cytoadhere *in vitro *and *in vivo*.

**Results:**

These comparisons revealed that only two *var *genes (*var1csa *and *var2csa*) are conserved in all three isolates and one *var *gene (Type 3 *var*) has homologs in IT4 and 3D7. While the remaining 50 plus genes in each isolate are highly divergent most can be classified into the three previously defined major groups (A, B, and C) on the basis of 5' flanking sequence and chromosome location. Repertoire-wide sequence comparisons suggest that the conserved homologs are evolving separately from other *var *genes and that genes in group A have diverged from other groups.

**Conclusion:**

These findings support the existence of a *var *gene recombination hierarchy that restricts recombination possibilities and has a central role in the functional and immunological adaptation of *var *genes.

## Background

Malaria pathogenesis poses a major hindrance to development in many parts of the world with more than 500 million people suffering from the disease and at least one million dying from *P. falciparum *infection each year [1]. Disease severity has been associated with the accumulation of infected erythrocytes (IEs) in microvasculature of vital organs, such as the brain and placenta [2]. A key protein family involved in IE binding is the antigenic variant *P. falciparum *Erythrocyte Membrane 1 (PfEMP1) [3-5]. Each parasite genome contains about 60 *var *genes that encode PfEMP1 proteins [6], which are expressed in a mutually exclusive fashion at the IE surface [7,8]. Switches in *var *gene expression allow parasites to evade the host antibody response and sequester at different microvascular sites in the body [9]. Therefore, further definition of the *var *gene family conservation, the factors regulating variant antigen gene diversification, and the expression of particular *var *genes during disease will provide critical insights into malaria pathogenesis and aid disease interventions.

*Var *genes have a two-exon structure [4]. The first exon is large (~3.5 to 9.0 kb) and encodes multiple adhesion domains called the Duffy binding-like (DBL) and cysteine-rich interdomain region (CIDR). The second exon is smaller (~1.0 to 1.5 kb) and codes for a more conserved cytoplasmic tail. Although PfEMP1 sequences are highly diverse, the adhesion domains can be grouped by sequence similarity [10] into seven types of DBL domains (α, α_1_,β, γ, δ, ε, and x) and four types of CIDR domains (α, α_1_, β, and γ) that have been used as criteria for dissecting PfEMP1 protein domain structures and binding functions.

The PfEMP1 proteins in the 3D7 genome have been arbitrarily classified into one of seventeen different protein architectural types based upon domain composition [6,11] and divided into three major (A, B, and C) and two intermediate (B/A and B/C) groups on the basis of 5' upstream (Ups) sequence and chromosomal location [6,12,13]. *Var *group A genes have UpsA flanking sequences and are located in sub-telomeric regions transcribed toward the telomere, while group B consists of telomeric *var *genes flanked by UpsB sequences that are transcribed toward the centromere, and group C are flanked by UpsC sequences and are located in central chromosomal regions. Group B/A genes are very similar in location and transcriptional orientation to group B genes, but are located further from the telomere following other *var *genes or pseudogenes. In contrast, group B/C genes have an UpsB-like 5' flanking sequence, but are located in central chromosomal regions. Thus, it has been postulated that groups B/A and B/C represent transitional groups between the major groupings [13].

Inter-isolate comparisons have also revealed the existence of three unusual genes: *var1csa, var2csa*, and Type 3 *var *genes, which appear in nearly all parasite isolates [12,14-19]. These semi-conserved homologs may have important roles in the host-parasite interaction. The PfEMP1 encoded by *var2csa *binds the placental adhesion receptor, chondroitin sulfate A (CSA), and therefore has a critical role in the pathogenesis of pregnancy associated malaria [20,21], while no function has yet been ascribed to the proteins encoded by *var1csa *and Type 3 *var*.

The genomic organization of *var *genes may have an important role in *var *gene evolution. Similar to other variant antigen families, gene recombination or gene conversion between *var *paralogs may contribute to the rapid evolution of the gene family [22-24]. It has been hypothesized that the frequency of recombination between *var *genes may depend upon chromosomal location, gene orientation, and homology in the gene flanking sequence. Sequence and binding analysis of 3D7 *var *genes indicate that groups B and C PfEMP1 proteins bind the primary microvasculature receptor (CD36) while group A PfEMP1 proteins do not [12,13,25]. Thus, *var *gene recombination hierarchies may promote the evolution of PfEMP1 adhesion groups with different patterns of sequestration and disease. A fundamental question is whether the gene organization observed in 3D7 occurs in other parasite isolates and contributes to a general recombination mechanism shaping the variant antigen repertoire.

To investigate evolutionary mechanisms of the *var *gene family and provide new tools to study the role of PfEMP1 proteins in mediating cytoadhesion, we have sequenced *var *genes from isolate IT4/25/4 (IT4), which has maintained the ability to cytoadhere after *in vitro *adaptation [26-29], and compared these genes to the *var *repertories of the 3D7 genome reference isolate and of the HB3 isolate, for which sequence contigs were recently made available (*Plasmodium falciparum *HB3 Sequencing Project, Broad Institute of Harvard and MIT [30]). Although there are currently relatively few reports, isolate HB3 also maintains cytoadherence in culture [31,32] and is therefore a useful addition to comparative *var *analyses.

All three parasites, IT4, HB3, and 3D7, have been cloned *in vitro *and represent single parasite genotypes. The IT4 parasite was originally isolated from Brazil [33], but is known to have undergone accidental cross-contamination at an early stage of its history after *in vitro *adaptation [34]. The HB3 clone was derived from the Honduras I/CDC isolate [35] and the NF54 parent to the 3D7 clone was isolated from an individual who lived near an airport in Amsterdam and never left the Netherlands [36]. Based upon genotyping and parasite population studies, IT4 groups with Asian isolates, 3D7 groups with African isolates, and HB3 represents Central America [37].

Despite progress in understanding the mechanisms of cytoadhesion and antigenic variation of PfEMP1, limited information about the factors regulating variant antigen diversification and the extent of repertoire overlap between parasite isolates exists. Most studies have relied on small *var *sequence "tags" amplified from the first DBL domain in PfEMP1 proteins [19,23,38-48]. The studies presented here represent the first comprehensive analyses of *var *genes across multiple parasite isolates. These comparisons reveal general principles of *var *gene organization that have become established across geographically diverse parasite isolates and provide powerful tools to study the cytoadherent and immunogenic properties of PfEMP1 proteins.

## Results

### The var gene repertoires from the IT4 and HB3 isolates

**Figure 1:**
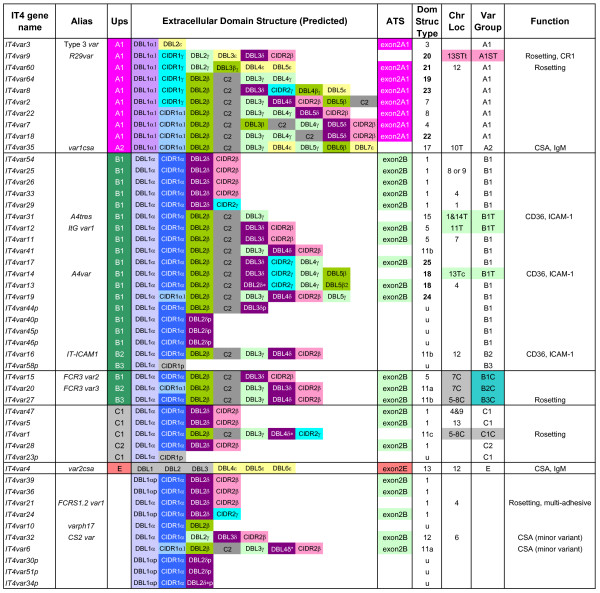
**Schematic representation of the IT4 *var *gene repertoire**. Gene names, Ups sequence type, domain architecture, chromosomal location, transcription orientation, and binding functions are listed. IT4 *var *genes are primarily assigned to different groups on the basis of 5' flanking sequence (Ups type) and chromosomal location when known. PfEMP1 proteins are comprised of multiple domains termed N-termimal segment (NTS), Duffy binding-like (DBL), cysteine-rich interdomain region (CIDR), C2, transmembrane (TM), and acidic terminal segment (ATS or exon2) which have been classified by sequence criteria into different types. The PfEMP1 proteins in the 3D7 clone were arbitrarily classified into 17 different protein architectural types on the basis of domain composition [6]. Types 18–25 (bolded) are unique to IT4. Chromosome locations are indicated as T, ST, SST: first, second, and third *var *genes from the telomere respectively. C: internal *var *genes. t: transcribed towards telomere, c: transcribed towards centromere. The chromosomal location of *var2csa *was determined in [78]. Accession numbers for newly sequenced genes are EF158071-EF158105.

Conservation in the *var *gene 5' and 3' gene-flanking regions, the semi-conserved exon 2, and other domains allowed us to design a series of primers (Additional file 4: Table S1) and extend IT4 *var *tags that we had previously sequenced from the PfEMP1 DBLα, β, γ, and δ domains [49]. We sequenced 28 full-length *var *genes and 10 partial genes from the IT4 isolate [GenBank:EF158071-EF158105], in addition to the 10 full-length *var *genes that have been previously characterized (Figure 1). These genes represent all but 11 of the 59 IT4 sequence tags identified from other studies (Tables S2 and S3)[42,49-51]. In order to estimate the proportion of IT4 *var *genes represented by these sequences, we searched the 1× coverage IT4 genome sequence at the Wellcome Trust Sanger Institute [52] for additional *var *sequences. Out of 949 reads with sequence similarity to the first exon of any known *var *genes, only ~15% do not overlap with our data set. Assembly of these reads shows that most of the non-overlapping reads represent small sequence fragments no larger than a single read and three partial gene fragments of 3–4 kb (data not shown). Thus, the *var *gene repertoire presented here includes partial or complete sequence for most IT4 *var *genes. Eight *var *genes were mapped to specific chromosomes using pulsed-field gel electrophoresis and Southern analysis (Figure 1, data not shown) and in some cases, intrachromosomal location (central versus sub-telomeric) was identified based on *Apa*I restriction fragment length [53]. The chromosomal locations of a further 13 *var *genes were based on previously published data [42].

**Figure 2:**
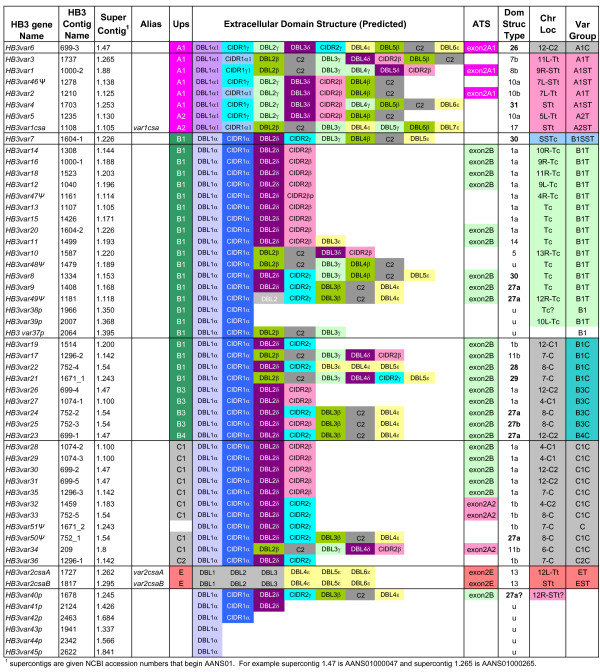
**Schematic representation of HB3 *var *genes**. Genes are organized as in figure 1 and grouped according to 5' flanking sequence (Ups type) and chromosomal location. Partial (p) and pseudogenes (Ψ) are labeled. Bolded domain structure types are unique to the HB3 parasite line. Binding properties have not been mapped to HB3 PfEMP1 proteins.

Analysis of the HB3 sequence contigs obtained from the 10× coverage genome sequence at the Broad Institute[30] identified 52 *var *genes that contain a DBLα domain as well as two *var2csa *homologs; 39 of the 54 *var *genes are full-length, 9 are incomplete and six are pseudogenes containing stops or frame-shifts (Figure 2). Examination of 5' and 3' flanking sequences (see Materials and Methods) enabled us to predict the chromosomal location of most genes (Figure 2), although in a number of cases they could not be assigned to specific chromosome ends. These predictions assume that recombination has not changed the arrangement of chromosome ends in HB3 and 3D7.

Comparisons of the IT4 and HB3 PfEMP1 protein domain architecture revealed representatives of most classes previously described in 3D7 (Additional file 1) [6] plus fourteen new types (Figures 1 &2). Of the 31 domain architectures, most types contain only a single representative per isolate and only seven (1, 5, 7, 8, 11, 13, and 17) are found in all three isolates. While five (2, 6, 9, 14, and 16) are found only in 3D7, eight (18–25) are unique to IT4, and six (26–31) to HB3 (Table 1). Moreover, the distribution of *var *genes among the shared domain architecture classes differs substantially between isolates. More than half (40/62) of the 3D7 *var *genes have a Type 1 architecture, but this type of *var *gene is rarer in IT4 (12/48) and HB3 (20/54). Conversely, IT4 contains six Type 11 *var *genes compared to only one in 3D7 and HB3; while HB3 contains six representatives of Type 27, which is not present in either 3D7 or IT4. The differential abundance of individual PfEMP1 types between parasite isolates and the presence of new PfEMP1 types in isolates IT4 and HB3 indicate considerable inter-strain plasticity in the variant antigen repertoire. Despite these differences in gene repertoires, the previously described tandem domain associations (DBLα-CIDR1, DBLβ-c2, and DBLδ-CIDR non-α types)[6,10] are consistently preserved, indicating the potential structural and functional significance of these domain relationships.

**Table 1:** *Var *gene chromosomal locations and domain architectures across isolates.

***Ups *group**	**Location**	**Domain architecture**		
		**3D7**	**IT4**	**HB3**
A1-2	STt	2, 3(3), 4, **7**, **8**^a^, 9, 10	3, 4, **7**, **8**, 19, 20, 21, 22, 23	**7**, **8**, 10(1+ψ), 31
	Cen			26
B1	Tc, STc	**1**(26), 2^b^, 6^b^, **11**^b^, 12, 14, 16	**1**(5), 5(3), **11**, 15, 18(2), 24, 25, P(4)	**1**(8+ψ), 5, **11**, 14, 27(1+ψ), 30(2)^b^, P(3+ψ)
	Cen	1		28, 29
B2-4	Tc, STc	1, 2^b^	11(3), P	
	Cen	1(2), 5, 15		1(2), 27(2+ψ)
C1		**1**(11), 5	**1**(2), 11, P	**1**(7), 11
C2	Cen	**1**	**1**	**1**
A2	STt	**17(ψ)**	**17**	10, **17**
E	STt	**13**	**13**	**13(2)**
Unclassified			1(4), 11, 12, P(4)	1(ψ), 27, P(6)

**Total**		**61+1ψ**	**48**^**c**^	**48+6ψ**^**c**^

^a^Bolded protein architectures were detected in all three isolates.

^b^These genes were members of the B-ST *var *group.

^c^The IT4 and HB3 are partial gene repertoire estimates.

While seven protein architectural types are shared among the three isolates, most *var *genes have overall amino acid sequence identities of < 50% in individual domains (Additional file 2), even those within the same architectural type. However, three *var *genes (*var1csa*, *var2csa*, and Type 3 *var*) are highly conserved at the sequence level, with > 75% identity over multiple domains. Partial gene sequence tags for these three *var *genes have been amplified from many parasite isolates indicating their unusual conservation for the *var *gene family [19]. However, these isolate-transcendent members can have different copy numbers between parasite isolates. For instance, while 3D7 has three copies of the Type 3 *var*, we could amplify only one copy in IT4 and did not find any copies in the genomic sequence from HB3. In addition, HB3 contains two *var2csa *copies rather than one copy as in 3D7 and IT4. Although *var1csa *is present in all three parasites, it is a truncated pseudogene in 3D7 (first exon) and the second exon has a frameshift in the HB3 allele. Also, *var1csa *is believed to be truncated in many field isolates [18] and has a distinct gene transcription pattern from other *var *genes [54]. Therefore it may have a different biological role than other *var *genes.

**Figure 3:**
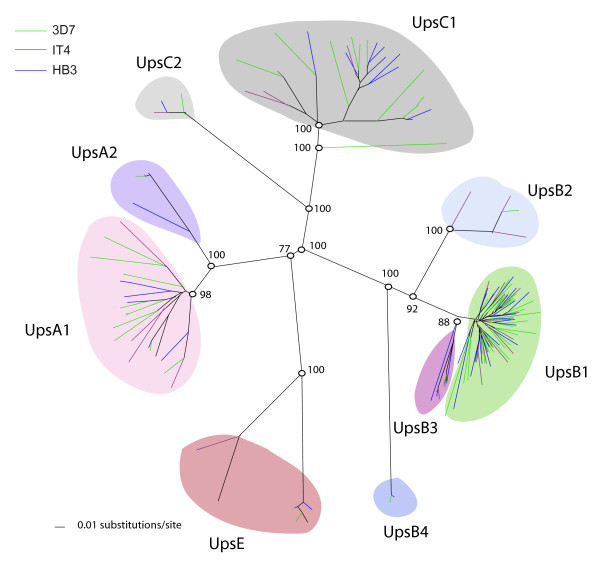
**Phylogenetic comparison of *var *gene flanking regions from IT4, HB3, and 3D7 parasite isolates**. A neighbor-joining tree was generated based upon 500 bp of 5' gene flanking sequence. Upstream groupings (Ups groups) with bootstrap support out of 1000 replicates are color shaded and labeled. Gene names have been removed from the figure for simplification.

Sequence comparison of 1.5–2.0 kb of 5' flanking sequence from the 3D7 *var *genes has defined five upstream types; UpsA, B, C, D, and E types [6,11-13]. Phylogenetic analysis of 500 bp of 5' flanking sequences from the IT4, HB3, and 3D7 *var *genes revealed that IT4 and HB3 have similar sequence groupings as 3D7 (Figure 3). While we found similar classes as in previous studies, we have sub-divided UpsB into four sub-groups (B1–B4) and UpsC into two sub-groups (C1 and C2). This study also revealed that UpsD is very similar to UpsA (Figure 3), and that these categories can be more accurately referred to as UpsA1 (formerly UpsA) and UpsA2 (formerly UpsD). Notably, the proportion of *var *genes in each Ups type is similar between isolates (Figures 1, 2, S1).

**Figure 4:**
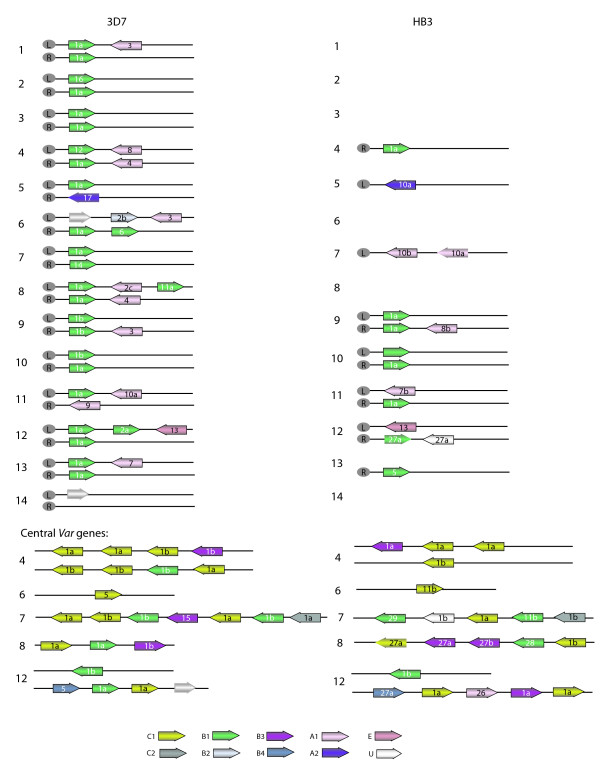
**Chromosomal distribution of *var *genes in the 3D7 and HB3 parasite isolates**. *Var *genes are color shaded according to 5' gene flanking Ups type (U indicates unknown) and labeled according to protein architecture. The chromosomal locations were predicted for 36 of the 54 HB3 PfEMP1 proteins based upon gene flanking sequence and comparison to the 3D7 reference genome (see methods). Arrows without an outline indicate pseudogenes.

The 3D7 *var *gene repertoire has been previously categorized into three major (A, B, and C) and two intermediate (B/A and B/C) groups on the basis of Ups sequence and chromosomal location [6,12,13]. The HB3 and IT4 *var *genes can be similarly assigned to the three major groups on the basis of Ups sequence (Figure 3), but differences in chromosomal location between isolates argue for a modification of the sub-groupings. For example, the HB3 repertoire contains one UpsA1-associated *var *gene (*HB3var6*) that is in a central chromosomal cluster rather than the typical sub-telomeric location (Figure 4). Therefore, to allow for the future addition of 'atypical' genes, we have developed a naming system based upon *var *gene location and Ups sequence type. The Ups types (A1-2, B1-B4, C1-2, and E) when known are listed first followed by a chromosome location reference. T, ST, and SST refer to the first, second, and third *var *genes from the telomere respectively and C refers to central *var *genes. For example, we have now separated *var *group A into sub-groups A1C, A1ST, and A1SST to represent central and sub-telomeric *var *genes, respectively. Similarly, group B is divided to represent *var *genes with corresponding central (B1C, B2C, etc.) or telomeric (B1T, B2T, etc.) locations. Members of the B/A group previously defined by Lavstsen *et al*. [13] are now classified as B1ST, B2ST, etc. denoting both the 5' upstream type plus a distinct sub-telomeric chromosomal location which follows other *var *genes or pseudogenes [6].

As observed previously [55], the *var *gene chromosomal location was highly predictive of 5' gene flanking sequence (Figure 4). For instance, nearly all centromere-transcribed *var *genes in the telomeric location were UpsB1 type (Figure 4). In contrast, members of the "transitional" B/C *var *group located in central chromosomal locations, associate with any of the 5' flanking sequences UpsB1-4. Interestingly, while HB3 contains a copy of the semi-conserved *var1csa *gene (domain architecture Type 17) with the expected UpsA2 sequence (formerly UpsD), the HB3 isolate is unique in having a second distinct PfEMP1 protein associated with the UpsA2 sequence (*HB3var4*, domain architecture Type 10). Thus, we have classified both within group A2ST. The highly conserved, and sequence divergent, *var2csa *remains in a separate Ups group (ET, EST, or ESST).

Although the general chromosomal distribution of *var *genes in sub-telomeric regions or central regions on chromosomes 4, 6, 7, 8, and 12 are similar between the three isolates, the genes themselves are not conserved with the exception of *var1csa*, *var2csa *and Type 3 *var*. Significantly,*var *genes in the same chromosomal location from the three isolates differ in both sequence and protein architecture (Figure 4, Additional file 4: Table S4). Furthermore, the order of *var *Ups types in central *var *gene clusters differ between isolates (Figure 4). These differences between isolates are evidence of gene recombination that has occurred within the coding and gene-flanking regions.

### Var gene recombination

**Figure 5:**
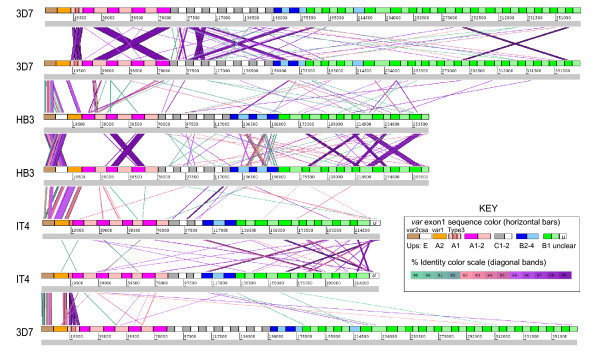
**ACT nucleotide comparison of *var *gene repertoires**. Concatamers of *var *gene exon1 sequences were arranged sequentially by Ups type: UpsE, A, C, B2-4, and B1, (colored as indicated) with one genome per horizontal line. The isolate-transcendent *var *genes *var2csa*, *var1csa *and Type 3 *var *are positioned at the left end of the concatemer. BLASTN was performed with word length set at 90 nucleotides (filter for low complexity removed). The comparisons were viewed in ACT with a window size of 120 nucleotides, at minimum 90% identity to show segments of similarity between *var *genes. Diagonal bands connecting individual *var *genes are colored according to percent identity, shown in the scale diagram (inset). Band width corresponds to region of sequence identity. *Var *names are listed, in order of appearance, in Additional file 4: Table S7.

To study the genetic relationship of different *var *genes, we performed repertoire-wide nucleotide and amino acid sequence comparisons using a number of different approaches and visualization tools (see Materials and Methods). The Artemis Comparison Tool (ACT) [56] was used to visualize regions of similarity identified by reciprocal BLASTN searches of *var *exon1 nucleotide sequences. Using criteria of a word size of 90nt, > 90% identity we observed the gene duplication in 3D7 (*PFD1235w *and *MAL8P1.207*) and identified one gene duplication in HB3 (*var2csaA *and *var2csaB*). These gene pairs are nearly identical over their entire lengths (Figure 5 & Additional file 4: Table S5). These analyses also visualize the semi-conserved *var *genes (*var1csa*, *var2csa *and Type 3 *var*) identified above, which have multiple regions of high sequence similarity (> 90%) between isolates (Additional file 4: Table S5).

**Figure 6:**
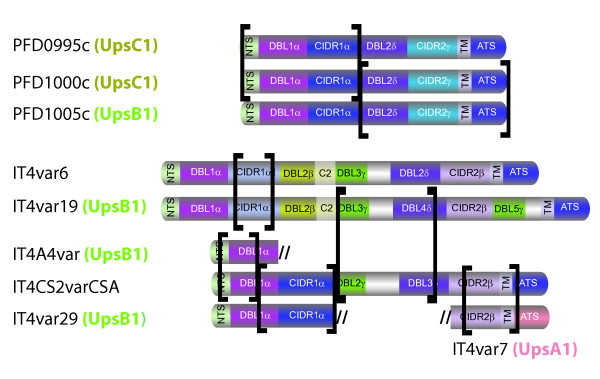
**Examples of chimeric genes in the 3D7 and IT4 parasite isolates**. Identical or nearly identical regions are indicated by brackets.

ACT comparisons also identified several instances of partial sequence similarity (greater than 500 bp) between two *var *genes of the same isolate. Selected examples are shown (Table 2), illustrating the segmental nature of sequence similarity between genes, with only part of each sequence showing a high degree of sequence similarity to the other partner(s). The 3D7 repertoire contains four examples of such "chimeric" gene pairs, the current set of IT4 *var *genes has eight, and the HB3 repertoire has six (Table 2). In some cases (*e.g. PFD0995c*/*PFD1000c*/*PFD1005c *in 3D7), a *var *gene appears to be a "true" chimera of two different *var *genes (Figure 6), while in other cases the chimeras represent partial duplications between two *var *genes.

**Table 2:** High scoring BLASTn matches within and between isolates.

Comparison isolates	var1	var2	Chromosome/group var1	Chromosome/group var2	% ID	Match length (nt)
**between**						
*HB3x3D7*	*HB3var2*	*MAL8P1.207*^a^	7/A1ST	8/A1ST	*93.62*	*1175*
*HB3x3D7*	*HB3var2*	*PFD1235w*^a^	7/A1ST	4/A1ST	*93.62*	*1175*
HB3x3D7	HB3var4	*PF13_0003*	A1ST	13/A1ST	91.19	636
HB3x3D7	*HB3var22*	*PF11_0521*	8/B1C	11/A1ST	92.62	664
HB3x3D7	*HB3var23*	*PFL1950w*	12/B4C^b^	12/B4C	99.59	972
ITxHB3	*IT4var28*	*HB3var35*	UpsC2	7/C1C	99.34	1057
ITxHB3	*ITvar16*	*HB3var37(inc)*	UpsB2	UpsB1	97	661
ITxHB3	*FCR3S1.2_1*	*HB3var27*	unknown	B3C	99	1703
3D7xIT	*PFD0020c*	*IT4var6*	4/A1ST	unknown	90	536
						
**within**						
3D7x3D7^c^	*PFD0635w*	*PFD0630c*	4/B3C	4/C1C	99	703
3D7x3D7	*PFD0635w*	*PFD0630c*	4/B3C	4/C1C	99.78	3645
3D7x3D7	*PFD1000c*	*PFD0995c*	4/C1C	4/C1C	100	2831
3D7x3D7	*PFD1005c*	*PFD1000c*	4/B1C	4/C1C	99.92	2551
3D7x3D7	*MAL13P1.1*	*PFE0005w*	13/B1T	5/B1T	100	2776
HB3xHB3	*HB3var3*	*HB3var4*	11/A1ST	A1ST	100	4948
HB3xHB3	*HB3var23*	*HB3var24*	12/B3C	8/B4C	94.52	1240
HB3xHB3	*HB3var23*	*HB3var24*	12/B3C	8/B4C	93.12	567
HB3xHB3	*HB3var26*	*HB3var23*	12/B3C	12/B3C	96.56	552
HB3xHB3	*HB3var25*	*HB3var24*	8/B3C	8/B4C	92.86	518
HB3xHB3	*HB3var15(inc)*	*HB3var14*	B1T	10/B1T	100	788
HB3xHB3	*HB3var15(inc)*	*HB3var14*	B1T	10/B1T	99.76	1657
HB3xHB3	*HB3var20*	*HB3var14*	B1T	10/B1T	100	2577
HB3xHB3	*HB3var7*	*HB3var8*	B1ST	B1T	99.86	5016
ITxIT	*CS2*	*IT4_var7*	6/unknown	UpsA1	99.67	905
ITxIT	*IT4var41*	*IT4_var16*	UpsB1	12/UpsB2	97.58	2359
ITxIT	*CS2*	*IT4_var19*	6/unknown	UpsB1	99.28	2221
ITxIT	*CS2*	*IT4_var29*	6/unknown	1/UpsB1	99.63	804
ITxIT	*CS2*	*IT4_var29*	6/unknown	1/UpsB1	93.69	697
ITxIT	*CS2*	*A4varICAM*	6/unknown	13/B1T	100	777
ITxIT	*FCR3S1.2_1*	*IT4var58*	4/unknown	unknown	98	1046
ITxIT	*IT4var23*	*IT4var58*	unknown	unknown	99	615
ITxIT	*IT4var6*	*IT4var19*	unknown	UpsB1	92	1348

^a^*MAL8P1.207 *and *PFD1235w *are duplicated in 3D7, so the match with *HB3var2 *effectively occurs only once.

^b^These two genes have a unique UpsB4 type 5' flanking sequence and are the first genes in a chromosome 12 central cluster in HB3 and 3D7, respectively.

^c^Highlighted rows indicate multiple hits of high sequence similarity, shared between the same gene pairs.

Despite geographic separation of 3D7, IT4, and 3D7, similar examples of segmental sequence similarity greater than 500 bp can be seen between *var *genes of the different isolates; with five examples between 3D7 and HB3, three between HB3 and IT4 and one between 3D7 and IT4 (Table 2). Remarkably, one of the inter-isolate gene pairs, *HB3var23 *and *PFL1950w*, are both the first *var *genes in a central cluster on chromosome 12 (Figure 4). These two genes have nearly identical and highly distinctive UpsB4 type 5' flanking sequences (Figure 3) and share approximately 1000 bp of coding region identity (Table 2), but otherwise have diverged from one another. This region of similarity identifies a recombination event that likely predates the continental separation of *P. falciparum *isolates.

These analyses also demonstrate that most *var *genes share little sequence identity suggesting that the *var *genes have diverged extensively between parasite isolates and have undergone segmental recombination (Figure 5). However, the patterns of sequence identities are not random in that similarities preferentially occur between members of the same Ups group (Table 2). For instance, UpsA1 *var *genes are 7.3× more likely to share similarity with other UpsA1 genes than with different Ups groups and UpsB2-4 *var *genes are 8.6× more likely to share similarity within the UpsB2-4 group (Additional file 4: Table S6). The same trend holds for gene similarities involving smaller gene segments 90 nucleotides and up (Additional file 4: Table S6). An exception is central *var *genes, which contain "mixed" chimeras of UpsB and UpsC-associated *var *genes (Figure 6), suggesting both groups of central chromosome *var *genes are recombining.

**Figure 7:**
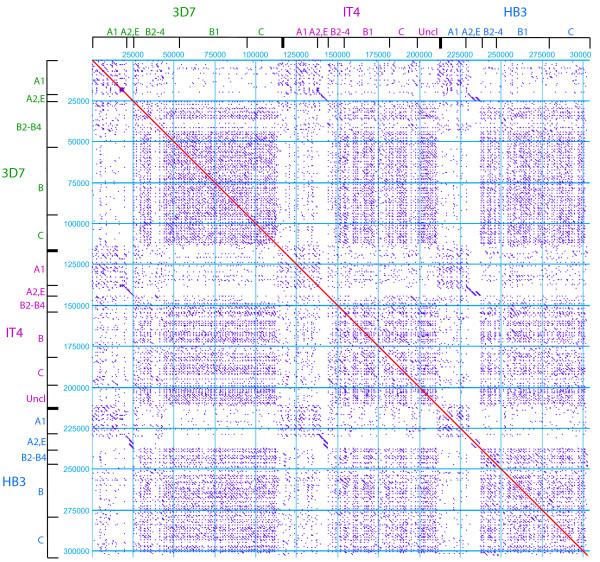
**Dotplot comparisons of PfEMP1 protein coding sequence**. The extracellular binding region of PfEMP1 proteins are organized by parasite isolate and 5' Ups sequence type. Uncl (unclassified) refers to sequences in which the Ups sequences have not been determined. Dot plot parameters include a window length of 30 amino acids and percent identity of 80% or greater.

To detect patterns of protein similarities, we conducted "repertoire-wide" dot-plot analyses using concatamers of *var *exon1 sequences ordered by isolate and 5' flanking sequence type. These analyses are designed to detect small windows of sequence similarity (80% amino acid identity, 30 amino acid window length) between PfEMP1 amino acid sequences and clearly show that UpsA PfEMP1 proteins share less similarity with UpsB and UpsC proteins than with other UpsA proteins (Figure 7). Conversely, UpsB and UpsC proteins are indistinguishable in terms of their degree of sequence identity with each other. This analysis combined with the analyses of individual domains (Additional file 2) shows approximately as much overall repertoire similarity within as between these geographically diverse strains.

To identify the regions of similarity between PfEMP1 proteins, the dotplot matches were plotted along the length of individual proteins. Overall, the DBL1 domains in PfEMP1 proteins tend to have the most similarity between proteins, although there are regions of similarity in some CIDR domains (Additional file 3). Most of the similarity between PfEMP1 proteins, including between the B and C groups, is associated with semi-conserved homology blocks in DBL domains (Additional file 3). These homology blocks correspond to structural elements in solved structures [57,58]. These analyses also clearly illustrate that *var2csa *and Type 3 proteins share almost no identity with other PfEMP1 proteins. Curiously, the rosetting-associated IT4var60 protein is not related to other UpsA proteins over most of its length (data not shown). However, unlike the semi-conserved Type 3 *var *or *var2csa*, HB3 and 3D7 do not have a *var60 *homolog. Although this result suggests that *IT4var60 *is not recombining with other *var *genes, more study is needed to determine its conservation in the parasite population. Taken together, these sequence comparisons support the hypothesis that *var *genes have differentiated into separately recombining groups that may be important to the evolution of the structure and function of PfEMP1 proteins.

## Discussion

While the sequencing of the 3D7 genome has contributed greatly in determining PfEMP1 functions and genetic diversity [reviewed in [11]], the associations of *var *gene repertoires within and between parasite isolates and the factors regulating variant antigen diversification remain largely unknown. To gain understanding into the evolutionary mechanisms shaping the variant antigen repertoire we present here the nearly complete *var *repertoire of a cytoadhesive laboratory isolate, IT4/25/5, and compare it to the 3D7 genome reference isolate and the recently sequenced HB3 genome.

Despite the enormous diversity of these genes, several features of the *var *gene family are conserved across isolates including *var *groupings based upon central or telomeric chromosome location and 5' flanking sequence that may have an important role in the evolution and function of *var *genes. It has been hypothesized that an original ancestral *var *gene was duplicated and diverged into the three main *var *groups (A, B, and C) and subsequently into additional transitional groups [13]. This interpretation is supported by our analyses showing similar categories of *var *genes in all three parasite isolates.

Based upon sequence comparisons, B and C groups are more similar, even though these genes tend to occupy different chromosomal locations at sub-telomeric and central chromosomal regions, respectively. However, the regions of similarity are predominantly associated with semiconserved homology blocks that are predicted to form the structural scaffolding for the DBL adhesion domains. Conversely, the group A genes differ greatly from the B and C groups while the coding region of the three isolate transcendent *var *genes, *var1csa*, *var2csa*, and Type 3 *var *genes have unique features and are different from all other *var *genes. However, these isolate transcendent *var *genes are more related to the UpsA group in that they are sub-telomeric, transcribed towards the telomere, and have 5' gene flanking regions that most resemble the UpsA type.

Repertoire-wide sequence comparisons show that most gene similarities occur between genes within the same *var *group, particularly for gene segments larger than 500 bp. An exception is central *var *clusters, which contain both UpsB and UpsC-associated *var *genes. While the functional significance of these different 5' promoter types is not completely understood [59], these two sets of central *var *genes appear to be recombining with each other. Taken together, these analyses suggest that *var *gene recombination preferentially occurs within *var *groups, with the exception of the semi-conserved *var *homologs that appear to recombine on their own. Further *var *gene comparisons of parasites undergoing more frequent recombination in nature or parasite crosses will be of interest to determine the relative frequency of intra- versus inter-group gene recombination. These findings provide insight into the mechanisms that generate antigenic diversity in *P. falciparum *through gene recombination hierarchies, and may have parallels in other variant antigen gene families in *Plasmodium *and other organisms.

The mechanisms of gene recombination/conversion are not well studied in *P. falciparum*. Sequence comparisons and restriction fragment length polymorphism analysis of parasite crosses and population studies suggest that both small (~100–200 nt) and larger recombination events contribute to *var *gene evolution [19,22,23]. Here, we observe that chimeric junction sites are often not "clean" breakpoints and have smaller sections of 90–95% identity 500 bp upstream and/or downstream of the central homologous region (data not shown). This feature may relate to a mechanism of recombination. Control of *var *gene expression has been connected to silence-inducing regulators of gene expression (e.g. Silent Information Regulator protein 2, SIR2) and chromatin packaging [60,61]. Recent studies have shown a possible link between factors involved in transcription regulation (including SIR2) and recombination (reviewed in [62,63]). It is interesting to speculate that in *P. falciparum*, factors that are silencing/controlling *var *gene expression may also be involved in the recombination and gene conversion mechanisms.

From a study of 3D7 *var *genes expressed after antibody selection, it has been hypothesized that group A may contain common antigenic types that are responsible for severe disease [64]. Although the duplicated 3D7 UpsA *var *genes, *PFD1235w *and *MAL8P1.207*, have been proposed as a fourth isolate-transcendent variant, termed *var4*, our analyses do not support this conclusion since a *var4 *homolog was not found in IT4 or HB3, although the HB3 isolate contained a match over a portion of the gene. In addition, *var4*-like gene fragments were not common in a global survey of parasite isolates using gene-specific primers [19]. Instead, this observation may represent one of a number of between-genome *var *chimeras which are not present in all parasite genomes. More study is required to determine which segments of *var4 *are maintained in the parasite population and the extent to which the same segments are shared by different parasite isolates.

More generally, with the exception of the Type 3 *var *genes and *var1csa*, the UpsA-associated *var *genes are not highly conserved between the three isolates. This observation reinforces findings of high genetic diversity of UpsA-associated DBLα tags from a global collection of parasite isolates [19]. Various factors may influence the stability of large *var *gene segments across a parasite population, including malaria endemicity, the frequency of mixed infections, or functional selection on that gene segment for binding. The diversity of the UpsA *var *genes suggests that antibody cross-reactivity between different parasite isolates does not necessarily imply the presence of isolate-transcendent *var *genes, but may be due to cross-reacting antibody epitopes on different PfEMP1 sequences. Although the possibility that a subset of *var *genes may be associated with severe malaria remains, these genes may not be as conserved across parasite isolates as the pregnancy malaria vaccine candidate *var2csa*.

The concept of a recombination hierarchy has implications for the evolution of parasite virulence and disease investigation. The conservation of *var *groupings across isolates raises the possibility that *var *groups may be diverging and/or evolving in characteristic patterns. For instance, group A *var *genes, with the exception of the Type 3 *var *genes, encode larger proteins with more complex domain compositions and have different protein head structures (DBL1-CIDR1 domains) from other *var *groups (Figures 1 &2). In contrast, the relatively small Type 1 proteins, which consist of four adhesion domains, are not associated with group A in any of the isolates. It has been suggested that immune selection can cause polymorphic antigens to self-organize into sets of non-overlapping variants within a population [65]. Increased frequency of inter-locus recombination or gene conversion may also act as a homogenizing force leading to the functional and structural specialization of different gene groups [25]. Interestingly, the proportion of small to larger PfEMP1 proteins and the distribution of PfEMP1 architectural types differed between isolates. Given the different selective pressures for binding and immune evasion, it may be to the parasite's advantage to have different sets of recombining genes [66,67]. These sets might include genes optimized to promote rapid parasite growth and transmission in the non-immune host, diversified genes that promote parasite transmission and persistence of infection in the face of higher levels of host immunity or organ-specific variants that expand parasite tropism to new host tissues, such as the placenta.

Unlike many isolates that have been adapted to *in vitro *cultivation, the IT4 genotype stably maintains the cytoadherent phenotype and therefore has become the primary model for this virulence determinant. CD36 binding, intercellular adhesion molecule 1 (ICAM-1) binding, and infected erythrocyte rosetting, or the binding of infected erythrocytes to uninfected erythrocytes, have been shown to reside in multiple different IT4 PfEMP1 proteins [for review, see [68]]. Although proteins that bind the same host receptor frequently use the same type of binding domain [11], the overall protein architectures are highly distinct. For instance, three ICAM-1 binding PfEMP1 proteins (A4tres, A4var, and IT-ICAM-1) all use DBLβ c2 domains but have different domain structure types and two rosetting PfEMP1 proteins, R29var [51] and FCRS1.2var1 [51,69] also have different domain structure types and bind different receptors on the erythrocyte surface. Our study completes the sequences for three additional IT4 *var *genes upregulated in rosetting parasite clones, which were previously identified by only their DBLα tag sequences (*IT4var1, IT4var27*, and *IT4var60*) [70]. Overall, the rosetting *var *protein structures are not highly related (Figure 1), although the DBL1α_1 _domain is similar between the UpsA-linked R29var and IT4var60 predicted proteins, which may be significant because DBL1α_1 _is an erythrocyte binding region in R29var [51]. In contrast, the other three rosetting-linked *var *genes (*IT4var27*, *IT4var1*, and *FCRS1.2var1*) have DBLα domains instead of DBLα_1 _domains and associate with Ups B, C, or unknown Ups sequence. While these comparisons suggest that rosetting PfEMP1 proteins are not restricted to particular *var *groups, further study is needed to determine whether rosetting proteins in the same *var *group use a similar constellation of erythrocyte receptors.

## Conclusion

A detailed understanding of the molecular mechanisms responsible for malaria pathogenesis is lacking, partly because of the complexity of the *var *gene family and the inability to model cytoadhesion with most culture-adapted laboratory isolates. In this study, we determined the *var *gene repertoires from the IT4 and HB3 isolates and provide evidence for a recombination hierarchy that shapes the evolution of the PfEMP1 virulence determinant. Furthermore, determination of the nearly complete *var *gene repertoire from the cytoadhesive IT4 parasite genotype, which has been adapted to both grow in the laboratory and infect new world monkeys, provides a unique capability to model cytoadhesion and immune acquisition *in vitro *and *in vivo*. Future binding and expression studies with cytoadhesive laboratory isolates, such as IT4 and HB3, in conjunction with analyses of the fully sequenced genomes will allow us to classify PfEMP1 proteins into biologically meaningful subsets and greatly accelerate understanding into malaria pathogenesis and immune evasion.

## Methods

### Parasites

*Var *genes were cloned from genomic DNA of the A4 clonal line. The A4 clone was originally derived by micromanipulation from *P. falciparum *isolate IT4/25/5 [29]. The IT4/25/5 isolate is one of several isolates including FVO, FCR3, and Palo Alto that appear to have a common genetic origin due to a laboratory cross-contamination event [34].

### Long PCR amplification of var gene sequences

Larger *var *gene PCR products were amplified from genomic DNA using previously described techniques [49]. PCR primers (Additional file 4: Table S1) were designed to the different types of *var *gene flanking sequence or the relatively conserved *var *exon 2. These primers were paired with gene-specific primers from small sequence tags that had been amplified from internal domains in IT4 PfEMP1 proteins [49]. PCR reactions were done using TaKaRa LA Taq™ polymerase (Fisher) following the manufacturer's recommendations and supplied buffer. 50 ul reactions containing 50 ng template, 1× buffer, 0.4 mM dNTPs (each), 2.5 mM MgCl_2_, 0.5 μM primers, and 2.5 U enzyme were run in a DNA Engine Dyad™ Peltier Thermal Cycler from MJ Research. PCR conditions were 1 cycle of 94°C for 1 min followed by 35 cycles of 98°C for 1 min, primer annealing temperature for 1 min, and an extension temperature of 62–68°C for 8–18 min. Primer annealing temperatures were 0–5 degrees lower the TMs listed in Additional file 4: Table S1. Sequencing was performed on PCR products that were directly hydosheared and cloned into a sequencing vector or were first cloned into the pCR^®^4-TOPO vector from Invitrogen before hydoshearing and subcloning into the sequencing vector. Sequences were assembled using the PHRED/PHRAP/CONSED software suite [71]. To confirm that recombination had not occurred during the PCR reaction or bacterial cloning, specific oligos were designed along the length of *var *genes and independent PCR reactions were performed on genomic DNA.

### Var gene chromosome assignation by pulsed field gel

IT4/FCR3 parasites were suspended in agarose blocks, then the intact chromosomes were size-fractionated on pulsed-field gels as described [72]. Gels were depurinated for 10 min in 0.25 M HCl, rinsed, then capillary transferred to Hybond N+ (GE/Amersham) in 0.4 M NaOH. Blots were hybridized at 50°C with DBLα tag probes corresponding to *var *genes *IT4var1*,*IT4var5*, *IT4var25*, *IT4var27*,*IT4var 29*,*IT4var33*, *IT4var60 *and *A4Tres*, as detailed previously [73]. Chromosome-central location of *IT4var1 *and *IT4var27 *was confirmed by ApaI digestion and size separation on pulsed-field gels, with hybridization to these tags at 60°C. Subtelomeric *var *genes lie on relatively short ApaI fragments [53], and these two genes are on large (> 400kbp) ApaI fragments.

### Extraction of var gene sequences from public genome project information

*Var *genes were identified in HB3 contigs downloaded from the *Plasmodium falciparum *HB3 sequencing project, Broad Institute of Harvard and MIT [30]. Contig assembly 1 was used, which contains approximately 10× genomic coverage. To identify *var *genes, sequences were searched for a common DBLα motif, DIGDI, using Artemis genome viewer (Rutherford et al 2000). *Var *genes retrieved in this manner were confirmed by comparison to results from BLASTN with the full DBL1α sequence of *PFA0005w*, at the Broad Institute malaria website. HB3 homologs for *var2csa *were identified by BLASTN search at the same website, using the 3D7 allele. HB3 *pseudovar *genes were not confirmed by reamplification, but had approximately the same level of sequence coverage as other *var *genes (8–10×). Where possible, the *var *gene chromosomal context was also noted, using %GC content graphs to locate both telomeres and GC-rich DNA elements, which are short sequences associated only with central *var *genes [74]. The predicted HB3 chromosomal assignments were based upon comparison to the 3D7 isolate using the NUCmer program in MUMmer to identify sequence identities in *var *gene flanking regions [75]. Sequence data for *P. falciparum *3D7 *var *genes and unassembled IT4 *var *reads were obtained from The Sanger Institute website [52]. Sequencing of *P. falciparum *IT4 is a component of the BioMalPar Consortium. For the ACT comparisons, two new 3D7 *var *genes are included, *MAL7P1.212 *and *MAL8P1.220*, which appear in the latest annotation [52]. These are both the most common type, Type 1, with UpsB type promoters, bringing the total number of this type in 3D7 up to 40 (out of 61 *var *genes).

### Sequence analysis

PfEMP1 domain classification was performed according to previous criteria [10]. Neighbor-joining trees for all of the domains and flanking regions were generated using ClustalX for multiple alignments and PAUP*4.0b10 (* Phylogenetic Analysis Using Parsimony and other methods) [76]. Bootstrap analysis was performed with 1000 replicates. Gap opening and gap extension penalties of 5.0 and 0.05 or 10 and 0.1 were used for amino acid and DNA alignments, respectively. Percentage sequence identities of DBL, CIDR, and C2 domains were calculated using the algorithm in DNAStar MEGALIGN, version 5.0 based upon a ClustalW alignment. Means and standard deviations of these percentages were calculated and plotted in Excel.

Dotplot analysis was performed on concatemers of the variable extracellular region (exon1) sequences ordered by isolate and Ups type using the programs Megalign and DSGene. A percent identity matrix was used for all parameters tested (window length and percent identity threshold). To visualize the alignment results at the level of individual proteins, the output alignments from the dotplot were collected, and for each alignment, the aa positions of the alignment and Ups category of the pair of proteins were determined. For each aa position, the number of "alignment hits" from each Ups category was counted and plotted along the length of individual proteins. Microsoft Excel was used to generate plots of the number of hits from 3D7 PfEMP1 proteins of the different Ups types along the length of individual proteins for both IT4 and 3D7 PfEMP1 proteins. Based upon the distribution of *var *genes in the 3D7 isolate, the maximum number of hits at individual amino acid positions for genes of each promoter type is UpsA (9), UpsB (22), UpsC (13), UpsA2 (formerly UpsD; 1), UpsE (1), and UpsB2-4 (13, based upon previous published 2000 bp tree) [6,12].

The Artemis Comparison Tool (ACT) was used to view exon1 for IT4, 3D7 and HB3 *var *genes. For each genome, a concatemer of *var *exon1 nucleotide sequences (from the start ATG to the splice donor site) was created. Sequences were organized by Ups group, and a string of 30 N's placed between each exon1 pair to clarify gene borders. Local BLASTN, (word length 90 nucleotides, filter for low complexity removed) was performed in all possible combinations between IT, 3D7 and HB3 *var *exon1 concatemers. These comparisons were then viewed with ACT [56,77], with different window sizes (90 to 510 nucleotides), 90% minimum identity, and self-matches removed.

## Authors' contributions

SMK carried out IT4 *var *gene sequencing, performed dotplot, phylogenetic, and other sequence analyses, and contributed to project design and coordination. SAK extracted *var *gene sequences from the HB3 public database and performed pulse-field gel analysis of IT4 *var *genes, ACT and sequence analysis of *var *genes. GA developed software to display dotplot information of individual proteins. ALS, SON, LMS, and WW amplified and sequenced IT4 *var *genes and performed PCR confirmations on genomic DNA. EL contributed to sequence analyses and ZC performed pulse-field gel analysis. CIN helped coordinate sequence analysis of *var *genes. PJM helped coordinate sequence analysis of *var *genes and performed the MUMmer analysis to assign HB3 *var *genes to chromosomes. JDS conceived the study, performed sequence analysis of *var *genes, and contributed to the projects design and coordination. SMK, SAK, CIN, PJM, and JDS wrote the manuscript. All authors approved the final manuscript.

## Supplementary Material

Additional file 4 Tables S1-S7 Click here for file

Additional file 1 Schematic representation of 3D7 *var *genes. Genes are organized as in Figures 1 and 2. Click here for file

Additional file 2 Amino acid identity of PfEMP1 adhesion domains from 3D7 and IT4 parasite isolates. Pair-wise comparisons were performed for all of the adhesion domains in 3D7 and IT4 PfEMP1 proteins. For each individual domain, the average identity to all other IT4 or 3D7 domains of that type was determined. These means were then averaged for 3D7 and IT4. Error bars represent one standard deviation of the mean of means. Click here for file

Additional file 3 Amino acid identity between PfEMP1 proteins of different Ups types. Alignment "hits" collected from the dot plot shown in Figure 7 were plotted per amino acid position of individual proteins. Graphs represent 3D7 PfEMP1 protein alignment "hits" (including the *var1csa *pseudo-gene) plotted against individual IT4 or 3D7 PfEMP1 proteins. Based upon the distribution of *var *genes in the 3D7 isolate, the maximum number of hits at individual amino acid positions for genes of each promoter type is UpsA (9), UpsB (22), UpsC (13), UpsA2 (formerly UpsD; 1), UpsE (1), and Ups B2-4 (13) [6,11]. Previously defined DBL homology blocks B, D and H are labeled [10]. Click here for file

## References

[B1] Snow RW, Guerra CA, Noor AM, Myint HY, Hay SI (2005). The global distribution of clinical episodes of *Plasmodium falciparum* malaria. Nature.

[B2] Miller LH, Baruch DI, Marsh K, Doumbo OK (2002). The pathogenic basis of malaria. Nature.

[B3] Baruch DI, Pasloske BL, Singh HB, Bi X, Ma XC, Feldman M, Taraschi TF, Howard RJ (1995). Cloning the *P. falciparum* gene encoding PfEMP1, a malarial variant antigen and adherence receptor on the surface of parasitized human erythrocytes. Cell.

[B4] Su XZ, Heatwole VM, Wertheimer SP, Guinet F, Herrfeldt JA, Peterson DS, Ravetch JA, Wellems TE (1995). The large diverse gene family var encodes proteins involved in cytoadherence and antigenic variation of *Plasmodium falciparum*-infected erythrocytes. Cell.

[B5] Smith JD, Chitnis CE, Craig AG, Roberts DJ, Hudson-Taylor DE, Peterson DS, Pinches R, Newbold CI, Miller LH (1995). Switches in expression of *Plasmodium falciparum* var genes correlate with changes in antigenic and cytoadherent phenotypes of infected erythrocytes. Cell.

[B6] Gardner MJ, Hall N, Fung E, White O, Berriman M, Hyman RW, Carlton JM, Pain A, Nelson KE, Bowman S, Paulsen IT, James K, Eisen JA, Rutherford K, Salzberg SL, Craig A, Kyes S, Chan MS, Nene V, Shallom SJ, Suh B, Peterson J, Angiuoli S, Pertea M, Allen J, Selengut J, Haft D, Mather MW, Vaidya AB, Martin DM, Fairlamb AH, Fraunholz MJ, Roos DS, Ralph SA, McFadden GI, Cummings LM, Subramanian GM, Mungall C, Venter JC, Carucci DJ, Hoffman SL, Newbold C, Davis RW, Fraser CM, Barrell B (2002). Genome sequence of the human malaria parasite *Plasmodium falciparum*. Nature.

[B7] Dzikowski R, Frank M, Deitsch K (2006). Mutually Exclusive Expression of Virulence Genes by Malaria Parasites Is Regulated Independently of Antigen Production. PLoS Pathog.

[B8] Voss TS, Healer J, Marty AJ, Duffy MF, Thompson JK, Beeson JG, Reeder JC, Crabb BS, Cowman AF (2006). A var gene promoter controls allelic exclusion of virulence genes in *Plasmodium falciparum* malaria. Nature.

[B9] Bull PC, Marsh K (2002). The role of antibodies to *Plasmodium falciparum*-infected-erythrocyte surface antigens in naturally acquired immunity to malaria. Trends Microbiol.

[B10] Smith JD, Subramanian G, Gamain B, Baruch DI, Miller LH (2000). Classification of adhesive domains in the *Plasmodium falciparum* erythrocyte membrane protein 1 family. Mol Biochem Parasitol.

[B11] Kraemer SM, Smith JD (2006). A family affair: var genes, PfEMP1 binding, and malaria disease. Curr Opin Microbiol.

[B12] Kraemer SM, Smith JD (2003). Evidence for the importance of genetic structuring to the structural and functional specialization of the *Plasmodium falciparum* var gene family. Mol Microbiol.

[B13] Lavstsen T, Salanti A, Jensen AT, Arnot DE, Theander TG (2003). Sub-grouping of *Plasmodium falciparum* 3D7 var genes based on sequence analysis of coding and non-coding regions. Malar J.

[B14] Fried M, Duffy PE (2002). Two DBLgamma subtypes are commonly expressed by placental isolates of *Plasmodium falciparum*. Mol Biochem Parasitol.

[B15] Rowe JA, Kyes SA, Rogerson SJ, Babiker HA, Raza A (2002). Identification of a conserved *Plasmodium falciparum* var gene implicated in malaria in pregnancy. J Infect Dis.

[B16] Salanti A, Jensen AT, Zornig HD, Staalsoe T, Joergensen L, Nielsen MA, Khattab A, Arnot DE, Klinkert MQ, Hviid L, Theander TG (2002). A sub-family of common and highly conserved *Plasmodium falciparum* var genes. Mol Biochem Parasitol.

[B17] Salanti A, Staalsoe T, Lavstsen T, Jensen AT, Sowa MP, Arnot DE, Hviid L, Theander TG (2003). Selective upregulation of a single distinctly structured var gene in chondroitin sulphate A-adhering *Plasmodium falciparum* involved in pregnancy-associated malaria. Mol Microbiol.

[B18] Winter G, Chen Q, Flick K, Kremsner P, Fernandez V, Wahlgren M (2003). The 3D7var5.2 (varCOMMON) type var gene family is commonly expressed in non-placental *Plasmodium falciparum* malaria. Molecular and Biochemical Parasitology.

[B19] Trimnell AR, Kraemer SM, Mukherjee S, Phippard DJ, Janes JH, Flamoe E, Su XZ, Awadalla P, Smith JD (2006). Global genetic diversity and evolution of var genes associated with placental and severe childhood malaria. Mol Biochem Parasitol.

[B20] Salanti A, Dahlback M, Turner L, Nielsen MA, Barfod L, Magistrado P, Jensen AT, Lavstsen T, Ofori MF, Marsh K, Hviid L, Theander TG (2004). Evidence for the involvement of VAR2CSA in pregnancy-associated malaria. J Exp Med.

[B21] Smith JD, Deitsch KW (2004). Pregnancy-associated malaria and the prospects for syndrome-specific antimalaria vaccines. J Exp Med.

[B22] Freitas-Junior LH, Bottius E, Pirrit LA, Deitsch KW, Scheidig C, Guinet F, Nehrbass U, Wellems TE, Scherf A (2000). Frequent ectopic recombination of virulence factor genes in telomeric chromosome clusters of *P. falciparum*. Nature.

[B23] Taylor HM, Kyes SA, Newbold CI (2000). Var gene diversity in *Plasmodium falciparum* is generated by frequent recombination events. Mol Biochem Parasitol.

[B24] Ward CP, Clottey GT, Dorris M, Ji DD, Arnot DE (1999). Analysis of *Plasmodium falciparum* PfEMP-1/var genes suggests that recombination rearranges constrained sequences. Mol Biochem Parasitol.

[B25] Robinson BA, Welch TL, Smith JD (2003). Widespread functional specialization of *Plasmodium falciparum* erythrocyte membrane protein 1 family members to bind CD36 analysed across a parasite genome. Mol Microbiol.

[B26] Biggs BA, Anders RF, Dillon HE, Davern KM, Martin M, Petersen C, Brown GV (1992). Adherence of infected erythrocytes to venular endothelium selects for antigenic variants of *Plasmodium falciparum*. J Immunol.

[B27] Bourke PF, Holt DC, Sutherland CJ, Kemp DJ (1996). Disruption of a novel open reading frame of *Plasmodium falciparum* chromosome 9 by subtelomeric and internal deletions can lead to loss or maintenance of cytoadherence. Mol Biochem Parasitol.

[B28] Day KP, Karamalis F, Thompson J, Barnes DA, Peterson C, Brown H, Brown GV, Kemp DJ (1993). Genes necessary for expression of a virulence determinant and for transmission of *Plasmodium falciparum* are located on a 0.3-megabase region of chromosome 9. Proc Natl Acad Sci U S A.

[B29] Roberts DJ, Craig AG, Berendt AR, Pinches R, Nash G, Marsh K, Newbold CI (1992). Rapid switching to multiple antigenic and adhesive phenotypes in malaria. Nature.

[B30] (2007). http://www.broad.mit.edu.

[B31] Johnson JK, Swerlick RA, Grady KK, Millet P, Wick TM (1993). Cytoadherence of *Plasmodium falciparum*-infected erythrocytes to microvascular endothelium is regulatable by cytokines and phorbol ester. J Infect Dis.

[B32] Xiao L, Yang C, Dorovini-Zis K, Tandon NN, Ades EW, Lal AA, Udhayakumar V (1996). Plasmodium falciparum: involvement of additional receptors in the cytoadherence of infected erythrocytes to microvascular endothelial cells. Exp Parasitol.

[B33] Udeinya IJ, Graves PM, Carter R, Aikawa M, Miller LH (1983). *Plasmodium falciparum*: effect of time in continuous culture on binding to human endothelial cells and amelanotic melanoma cells. Exp Parasitol.

[B34] Robson KJH, Walliker D, Creasey A, McBride J, Beale G, Wilson RJM (1992). Cross-contamination of Plasmodium cultures. Parasitology Today.

[B35] Bhasin VK, Trager W (1984). Gametocyte-forming and non-gametocyte-forming clones of *Plasmodium falciparum*. Am J Trop Med Hyg.

[B36] Ponnudurai T, Leeuwenberg AD, Meuwissen JH (1981). Chloroquine sensitivity of isolates of *Plasmodium falciparum* adapted to in vitro culture. Trop Geogr Med.

[B37] Mu J, Awadalla P, Duan J, McGee KM, Joy DA, McVean GA, Su XZ (2005). Recombination hotspots and population structure in *Plasmodium falciparum*. PLoS Biol.

[B38] Peterson DS, Miller LH, Wellems TE (1995). Isolation of multiple sequences from the *Plasmodium falciparum* genome that encode conserved domains homologous to those in erythrocyte- binding proteins. Proc Natl Acad Sci U S A.

[B39] Kyes S, Taylor H, Craig A, Marsh K, Newbold C (1997). Genomic representation of var gene sequences in *Plasmodium falciparum* field isolates from different geographic regions. Mol Biochem Parasitol.

[B40] Fowler EV, Peters JM, Gatton ML, Chen N, Cheng Q (2002). Genetic diversity of the DBLalpha region in *Plasmodium falciparum* var genes among Asia-Pacific isolates. Mol Biochem Parasitol.

[B41] Wunderlich G, Alves FP, Golnitz U, Tada MS, Camargo EF, Pereira-da-Silva LH (2005). Rapid turnover of *Plasmodium falciparum* var gene transcripts and genotypes during natural non-symptomatic infections. Rev Inst Med Trop Sao Paulo.

[B42] Fernandez V, Chen Q, Sundstrom A, Scherf A, Hagblom P, Wahlgren M (2002). Mosaic-like transcription of var genes in single *Plasmodium falciparum* parasites. Mol Biochem Parasitol.

[B43] Kirchgatter K, Mosbach R, del Portillo HA (2000). *Plasmodium falciparum*: DBL-1 var sequence analysis in field isolates from central Brazil. Exp Parasitol.

[B44] Afonso Nogueira P, Wunderlich G, Shugiro Tada M, d'Arc Neves Costa J, Jose Menezes M, Scherf A, Pereira-da-Silva LH (2002). *Plasmodium falciparum*: analysis of transcribed var gene sequences in natural isolates from the Brazilian Amazon region. Exp Parasitol.

[B45] Kaestli M, Cortes A, Lagog M, Ott M, Beck HP (2004). Longitudinal assessment of *Plasmodium falciparum* var gene transcription in naturally infected asymptomatic children in Papua New Guinea. J Infect Dis.

[B46] Bull PC, Berriman M, Kyes S, Quail MA, Hall N, Kortok MM, Marsh K, Newbold CI (2005). *Plasmodium falciparum* Variant Surface Antigen Expression Patterns during Malaria. PLoS Pathogens.

[B47] Taylor HM, Kyes SA, Harris D, Kriek N, Newbold CI (2000). A study of var gene transcription in vitro using universal var gene primers. Mol Biochem Parasitol.

[B48] Kirchgatter K, Portillo Hdel A (2002). Association of severe noncerebral *Plasmodium falciparum* malaria in Brazil with expressed PfEMP1 DBL1 alpha sequences lacking cysteine residues. Mol Med.

[B49] Kraemer SM, Gupta L, Smith JD (2003). New tools to identify var sequence tags and clone full-length genes using type-specific primers to Duffy binding-like domains. Mol Biochem Parasitol.

[B50] Duffy MF, Brown GV, Basuki W, Krejany EO, Noviyanti R, Cowman AF, Reeder JC (2002). Transcription of multiple var genes by individual, trophozoite-stage *Plasmodium falciparum* cells expressing a chondroitin sulphate A binding phenotype. Mol Microbiol.

[B51] Rowe JA, Moulds JM, Newbold CI, Miller LH (1997). *P. falciparum* rosetting mediated by a parasite-variant erythrocyte membrane protein and complement-receptor 1. Nature.

[B52] Wellcome Trust Sanger Institute. http://www.sanger.ac.uk/Projects/P_falciparum.

[B53] Thompson JK, Rubio JP, Caruana S, Brockman A, Wickham ME, Cowman AF (1997). The chromosomal organization of the *Plasmodium falciparum* var gene family is conserved. Mol Biochem Parasitol.

[B54] Kyes SA, Christodoulou Z, Raza A, Horrocks P, Pinches R, Rowe JA, Newbold CI (2003). A well-conserved *Plasmodium falciparum* var gene shows an unusual stage-specific transcript pattern. Mol Microbiol.

[B55] Voss TS, Thompson JK, Waterkeyn J, Felger I, Weiss N, Cowman AF, Beck HP (2000). Genomic distribution and functional characterisation of two distinct and conserved *Plasmodium falciparum* var gene 5' flanking sequences. Mol Biochem Parasitol.

[B56] Carver TJ, Rutherford KM, Berriman M, Rajandream MA, Barrell BG, Parkhill J (2005). ACT: the Artemis Comparison Tool. Bioinformatics.

[B57] Singh SK, Hora R, Belrhali H, Chitnis CE, Sharma A (2006). Structural basis for Duffy recognition by the malaria parasite Duffy-binding-like domain. Nature.

[B58] Tolia NH, Enemark EJ, Sim BK, Joshua-Tor L (2005). Structural basis for the EBA-175 erythrocyte invasion pathway of the malaria parasite *Plasmodium falciparum*. Cell.

[B59] Frank M, Deitsch K (2006). Activation, silencing and mutually exclusive expression within the var gene family of *Plasmodium falciparum*. Int J Parasitol.

[B60] Duraisingh MT, Voss TS, Marty AJ, Duffy MF, Good RT, Thompson JK, Freitas-Junior LH, Scherf A, Crabb BS, Cowman AF (2005). Heterochromatin Silencing and Locus Repositioning Linked to Regulation of Virulence Genes in *Plasmodium falciparum*. Cell.

[B61] Freitas-Junior LH, Hernandez-Rivas R, Ralph SA, Montiel-Condado D, Ruvalcaba-Salazar OK, Rojas-Meza AP, Mancio-Silva L, Leal-Silvestre RJ, Gontijo AM, Shorte S, Scherf A (2005). Telomeric Heterochromatin Propagation and Histone Acetylation Control Mutually Exclusive Expression of Antigenic Variation Genes in Malaria Parasites. Cell.

[B62] Verger A, Crossley M (2004). Chromatin modifiers in transcription and DNA repair. Cellular and Molecular Life Sciences (CMLS).

[B63] Lombard DB, Chua KF, Mostoslavsky R, Franco S, Gostissa M, Alt FW (2005). DNA Repair, Genome Stability, and Aging. Cell.

[B64] Jensen AT, Magistrado P, Sharp S, Joergensen L, Lavstsen T, Chiucchiuini A, Salanti A, Vestergaard LS, Lusingu JP, Hermsen R, Sauerwein R, Christensen J, Nielsen MA, Hviid L, Sutherland C, Staalsoe T, Theander TG (2004). *Plasmodium falciparum* associated with severe childhood malaria preferentially expresses PfEMP1 encoded by group A var genes. J Exp Med.

[B65] Gupta S, Maiden MC, Feavers IM, Nee S, May RM, Anderson RM (1996). The maintenance of strain structure in populations of recombining infectious agents. Nat Med.

[B66] Bull PC, Kortok M, Kai O, Ndungu F, Ross A, Lowe BS, Newbold CI, Marsh K (2000). *Plasmodium falciparum*-infected erythrocytes: agglutination by diverse Kenyan plasma is associated with severe disease and young host age. J Infect Dis.

[B67] Bull PC, Lowe BS, Kortok M, Marsh K (1999). Antibody recognition of *Plasmodium falciparum* erythrocyte surface antigens in Kenya: evidence for rare and prevalent variants. Infect Immun.

[B68] Kyes S, Horrocks P, Newbold C (2001). Antigenic variation at the infected red cell surface in malaria. Annu Rev Microbiol.

[B69] Chen Q, Heddini A, Barragan A, Fernandez V, Pearce SF, Wahlgren M (2000). The semiconserved head structure of *Plasmodium falciparum* erythrocyte membrane protein 1 mediates binding to multiple independent host receptors. J Exp Med.

[B70] Horrocks P, Pinches R, Christodoulou Z, Kyes SA, Newbold CI (2004). Variable var transition rates underlie antigenic variation in malaria. Proc Natl Acad Sci U S A.

[B71] Gordon D, Abajian C, Green P (1998). Consed: a graphical tool for sequence finishing. Genome Res.

[B72] Hinterberg K, Scherf A (1994). PFGE: improved conditions for rapid and high-resolution separation of *Plasmodium falciparum* chromosomes. Parasitol Today.

[B73] Kyes S, Pinches R, Newbold C (2000). A simple RNA analysis method shows var and rif multigene family expression patterns in *Plasmodium falciparum*. Mol Biochem Parasitol.

[B74] Hall N, Pain A, Berriman M, Churcher C, Harris B, Harris D, Mungall K, Bowman S, Atkin R, Baker S, Barron A, Brooks K, Buckee CO, Burrows C, Cherevach I, Chillingworth C, Chillingworth T, Christodoulou Z, Clark L, Clark R, Corton C, Cronin A, Davies R, Davis P, Dear P, Dearden F, Doggett J, Feltwell T, Goble A, Goodhead I, Gwilliam R, Hamlin N, Hance Z, Harper D, Hauser H, Hornsby T, Holroyd S, Horrocks P, Humphray S, Jagels K, James KD, Johnson D, Kerhornou A, Knights A, Konfortov B, Kyes S, Larke N, Lawson D, Lennard N, Line A, Maddison M, McLean J, Mooney P, Moule S, Murphy L, Oliver K, Ormond D, Price C, Quail MA, Rabbinowitsch E, Rajandream MA, Rutter S, Rutherford KM, Sanders M, Simmonds M, Seeger K, Sharp S, Smith R, Squares R, Squares S, Stevens K, Taylor K, Tivey A, Unwin L, Whitehead S, Woodward J, Sulston JE, Craig A, Newbold C, Barrell BG (2002). Sequence of *Plasmodium falciparum* chromosomes 1, 3-9 and 13. Nature.

[B75] Kurtz S, Phillippy A, Delcher A, Smoot M, Shumway M, Antonescu C, Salzberg S (2004). Versatile and open software for comparing large genomes. Genome Biology.

[B76] Swofford DL, Olson GJ, Waddell PJ, Hillis DM, Hillis DM, Moritz C and Mable BK (1996). Phylogenetic Inference. Molecular Systematics.

[B77] Rutherford K, Parkhill J, Crook J, Horsnell T, Rice P, Rajandream MA, Barrell B (2000). Artemis: sequence visualization and annotation. Bioinformatics.

[B78] Viebig NK, Gamain B, Scheidig C, Lepolard C, Przyborski J, Lanzer M, Gysin J, Scherf A (2005). A single member of the *Plasmodium falciparum* var multigene family determines cytoadhesion to the placental receptor chondroitin sulphate A. EMBO Rep.

