# Supplementary material for: Patterns of gene recombination shape var gene repertoires in Plasmodium falciparum: comparisons of geographically diverse isolates
Source: BMC Genomics. 2007 Feb 7;8:45. doi: 10.1186/1471-2164-8-45 (PMC1805758; doi:10.1186/1471-2164-8-45)
Supplement: Additional file 4 — Tables S1-S7 [file 1471-2164-8-45-S4.pdf]

Table S1. Sequences of 5' and 3' flanking primers

Primer name	3D7 chromosomal location	TM	Sequence 5' - 3'
**5' oligos**			
Tel4.2 (upsB, *upsC*)	Sub-teleomeric, *central*	48	TGATAGATARTATRGATAGAGAGA
UpsB 5.1(upsB)	Sub-telomeric	49	CRGAAGAASATATTTGYCTC
Cen2.1 (upsC, upsB)	central, sub-telomeric 1	44	ATATGRTAGATAATAYAGATAGA
UpsC5.1 (upsC)	central	44	GTGATAACYAYTATATMATATAC
var gr2 5p 5.1 (upsA)	Sub-telomeric	50	ATKTATTAYATTTGTTGTAGGTGA
Type3 5p 5.1	Sub-telomeric	46	GGATAAGTGATRACATAATRT
**3' oligos**			
exon2 3.2end		51	TTATATATYCYAYAYATCYSMTATAGG
exon2 3.3		54	CCADYTCTTCATAYTCACTTTC
3ptel type13.1 (downsB)	Sub-telomeric	45	GTTATACTRYTAATTACTACTTC
3ptel type23.1 (downsA)	Sub-telomeric	50	CAAATAATCAMATGTGTCAAAYAR

R = A,G Y = C,T M = A,C K = G,T W = A,T D = A,G,T

Table S2. IT4 *var* tags for partial and full-length genes

IT4 var	Alias	A4 DBLα[a]	ItG2 DBLα[b]	FCR3S1.2 DBLα[c]	R29R DBLα[d]	A4[e] DBLb	DBLg	DBLd	GenBank Accession whole or partial gene
var1		43	ee	52		b11	g9		AY248889
var2						b2, 9			
Var3	Type 3 var								
var 4	*var2csa*								
var5		25	dd				g13		AJ007941, FCR3-CD36 (partial)
var6		5	P	C27				d21	
var7		34			4	b1	g5	d1	
var8		27	F	45		b6			
var 9	*It-R29*	9	j		1				Y13402
var10 (p)[e]		15	s	50		b5			
var11		18		44		b12		d9	
var 12	*FCR3varT11-1*		d.1	4, 10	3				U67959, U31083(*ItG var*)
var13			bb	58			g12		
var 15	*FCR3 var2*	16	Q	3					L40609 (2nd gene in contig)
var16		17	aa	9p			g10		AY578326 (*IT-ICAM* or *FCR3-ICAM*)
var17		26		z		b10	g8		
var18							g2	d16	
var19			i	29		b14	g4	d22	
var 20	*FCR3var3*								L40609 (first gene in contig)
var21	*FCR S1.2 var1*			1					AF003473
var24				12				d4	
var25		32	b	49				d13	
var26		21	R						
var 27		14	cc	C27				d7	orthologous to Dd2var1 (L40608)
var28		22						d24	
var29		10			2			d2	
var30 (p)[f]		1	L	46				d20	
var31	*It A4tres*	13							AF193424
var 32	*It-CS2varcsa*	41	U					d23	AF134154, *CS2-CSA*
var33		31	E					d14	
var34 (p)				11[f]				d8	
var35	*FCR3var CSA*	8	k						AJ133811
var36		30	ff					d6	
var39		12	O	C22				d5	
var40 (p)				65				d10	
var41			C					d18	
var43 (p)								d25	
var44 (p)		11	H					d19	
var45 (p)		29		71					
var46 (p)		24						d27	U67960, *FCR3-T3-1*
var47				70				d28	
var51 (p)								d12	
var53 (p)		6							
var54		2	A					d11	
var55 (p)	*A4var*	41	U					d29	L42244, *A4varICAM*
var60		19							
var64			W						

[a] CAC41284-CAC41316
[b] [1]
[c] [2]
[d] [3]
[e] [4]
[f] (p) denotes partial gene sequenced

Table S3. Orphan Tags

Orphan	A4 DBLα[a]	ItG2 DBLα[b]	FCR3S1.2 DBLα[c]	chromosome location	notes
1		b2			
2		m	57	4	
3	38				
4			13	4	related to FCR3S1.2
5		n	6	9	
6	7		48	4	
7		c.1			
8		v			
9	20	y			
10	4				
11		x			89% identical to *IT4var28*

[a]CAC41284-CAC41316
[b][1]
[c][2]

Table S4. ***Var*** **Gene chromosomal locations and domain types**

Chromosome	Location	3D7	HB3	IT4
1	LTel	B1Tc(1a)-A1STt(3)		
1	RTel	B1Tc(1a)		
2	LTel	B1Tc(16)		
2	RTel	B1Tc(1a)		
3	LTel	B1Tc(1a)		
3	RTel	B1Tc(1a)		
4	LTel	B1Tc(12)-A1STt(8)		
4	Cen1	<C1C(1a)<C1C(1a)<C1C(1b)<B3C(1b)	[B3C(1a)>C1C(1a)>C1C(1a)>]	
4	Cen2	<C1C(1b)<C1C(1b)<B1C(1b)<C1C(1a)	C1C(1b)	
4	RTel	B1Tc(1)-A1STt(4)	B1Tc(1a)	
5	LTel	B1Tc(1a)	A2Tt(10a)	
5	RTel	A2Tt(17ψ)		
6	LTel	Tψ-B2STc(2b)-A1SSTt(3)		
6	Cen	<C1C(5)	C1C(11b)	
6	RTel	B1Tc(1a)-B1STc(6)		
7	LTel	B1Tc(1a)	A1Tt(10b)-A1SSTt(10aψ)	
7	Cen	<C1C(1a)<C1C(1b)<B1C(1b)<B3C(15)< C1C(1a)<B1C(1b)<C2C(1a)	B1C(29)-?C(1b)-C1C(1a) - B1C(11b)-C2C(1b)	B1C(1)-B1C(5)-B2C(11a)
7	RTel	B1Tc(14)		
8	LTel	B1Tc(1a)-A1STt(2c)-B1SSTc(11a)		
8	Cen	C1C(1b)>B1C(1a)>B3C(1a)>	C1C(27aψ)-B3C(27a)-B3C(27b)-B1C(28)-C1C(1b)	
8	RTel	B1Tc(1a)-A1STt(4)		
9	LTel	B1Tc(1b)	B1C(1a)	
9	RTel	B1Tc(1b)-A1STt(3)	B1C(1a)-A1STt(8b)	
	LTel	B1Tc(1b)	B1Tc(u)	[ST (17) on either Left or Right tel]
10	RTel	B1Tc(1a)	B1Cc(1a)	
11	LTel	B1Tc(1a)-A1STt(10a)	?-A1Tt(7b)	
11	RTel	A1Tt(9)	B1Tc(1a)	
12	LTel	B1Tc(1a)-STc(2a)-ESSTt(13)	?-ETt(13)	STc(5) L or R?
12	Cen1	B1C(1b)	B1C(1b)	
12	Cen2	B4C(5)-B1C(1a)-C1C(1a)-ψ	B4C(27a)-C1C(1a)-A1C(26)-B3C(1a)-C1C(1a)	
12	RTel	B1Tc(1a)	B1Cc(27aψ)-?STt(27a)	
13	LTel	B1Tc(1a)-A1STt(7)		B1Tc(18)-A1STc(20) L or R?
13	RTel	B1Tc(1a)-STψ	B1Tt(5)	
14	LTel	Tψ		
14	RTel			

Ltel: left telomere, Rtel: right telomere, Cen: Central chromosomal location
Ups types are listed first as A1-2, B1-4, C1-2, and E (when known)

T, ST, SST: first, second, and third *var* genes from the telomere respectively. C: internal *var* genes.
t: transcribed towards telomere, c: transcribed towards centromere
Ψ: pseudogene

Table S5 Homologs and duplicated genes in all genomes

isolates	var1	var2	Ups var1	Ups var2	% ID[B]	Match length[C]
**Duplicated gene**						
3d7x3d7	*PFD1235w*	*MAL8P1.207*	A1	A1	100	9441
**Type3 var**						
3d7x3d7	*PFF0020c*	*PFA0015c*	A1	A1	98.98	1866
3d7x3d7	*PFI1820w*	*PFA0015c*	A1	A1	99.41	1866
3d7x3d7	*PFA0015c*	*PFF0020c*	A1	A1	98.66	2020
ITx3D7	*PFA0015c*	*IT4var3*	A1	A1	98.6	2006
ITx3D7	*PFF0020x*	*IT4var3*	A1	A1	98.45	1866
ITx3D7	*PFI1820w*	*IT4var3*	A1	A1	99.3	1866
**var1csa**						
3d7xHB3	*HB3var1csa*	*PFE1640w-ps*	A2	A2	96.8	1909
HB3xIT	*FCR3var1csa*[A]	*HB3var1csa*	A2	A2	96.18	680
HB3xIT	*FCR3var1csa*[A]	*HB3var1csa*	A2	A2	96.76	4689
HB3xIT	*FCR3var1csa*[A]	*HB3var1csa*	A2	A2	94.3	2367
ITx3D7	*PFE1640w-ps*	*FCR3var1csa*	A2	A2	95.69	743
ITx3D7	*PFE1640w-ps*	*FCR3var1csa*	A2	A2	94.33	1657
**var2csa**						
3d7xHB3	*HB3var2csaA*	*PFL0030c*	E	E	92.34	522
3d7xHB3	*HB3var2csaA*	*PFL0030c*	E	E	93.03	932
3d7xHB3	*HB3var2csaA*	*PFL0030c*	E	E	96.08	1839
3d7xHB3	*HB3var2csaB*	*PFL0030c*	E	E	91.1	854
3d7xHB3	*HB3var2csaB*	*PFL0030c*	E	E	95.76	566
3d7xHB3	*HB3var2csaB*	*PFL0030c*	E	E	95.25	547
3d7xHB3	*HB3var2csaB*	*PFL0030c*	E	E	96.87	511
HB3xHB3	*HB3var2csaB*	*HB3var2csaA*	E	E	97.69	1298
HB3xHB3	*HB3var2csaB*	*HB3var2csaA*	E	E	93.74	671
HB3xHB3	*HB3var2csaB*	*HB3var2csaA*	E	E	96.09	1814
HB3xHB3	*HB3var2csaB*	*HB3var2csaA*	E	E	97.53	527
HB3xHB3	*HB3var2csaB*	*HB3var2csaA*	E	E	98.61	1799
HB3xIT	*IT4var4*	*HB3var2csaA*	E	E	93.26	742
HB3xIT	*IT4var4*	*HB3var2csaA*	E	E	95.57	1016
HB3xIT	*IT4var4*	*HB3var2csaA*	E	E	96.08	944
HB3xIT	*IT4var4*	*1817*	E	E	92.18	1253
HB3xIT	*IT4var4*	*1817*	E	E	95.1	1307
ITx3D7	*PFL0030c*	*IT4var4*	E	E	92.08	1754
ITx3D7	*PFL0030c*	*IT4var4*	E	E	94.26	592
ITx3D7	*PFL0030c*	*IT4var4*	E	E	92.31	754
ITx3D7	*PFL0030c*	*IT4var4*	E	E	95.98	547
ITx3D7	*PFL0030c*	*IT4var4*	E	E	94.65	1440

[A] Highlighted rows indicate multiple hits of high sequence similarity, shared between homologous (or duplicated) gene pairs.
[B]Percent identity for each match, from blastn (word length 90 nucleotides)
[C]Length of each match, in nucleotides

Table S6. BLASTn similarity between *var* groups

	A	B1	B2-4	C
**A**	7.3,5.9	0.4,0.7	0.0,2.3	0.0,0.6
**B1**		0.5,0.7	0.4,2.6	0.5,1.3
**B2-4**			8.6,4.2	2.6,2.8
**C**				0.4,1.7

Observed/expected (O/E) ratios of BLASTn similarities were calculated for the different *var* groups. The first number in each cell represents the O/E ratio for gene similarities greater than 510 nucleotides (n = 42) and the second number represents O/E ratio for gene similarities greater than 90 nucleotides (n = 183). Expected values were determined from the number of genes in each group assuming random likelihood of BLASTn similarity between any two genes. Type 3 *var* genes and *var1csa* were excluded from these calculations because these genes only had similarity to their homologs in other isolates.

Table S7

Order and color-coding of var exon1 sequences in ACT diagram, with Ups type (Ups) and Chromosomal location/direction of transcription, where known [Location: T, first gene after telomere associated repeats; ST, subtelomeric (not first open reading frame on chromosome, or pseudogene between *var* and telomere repeats); Cen, chromosome-central; t, transcribed towards telomere; c, transcribed towards centromere].

ACT order	3D7	Ups	Location	ACT order	IT4	Ups	Loc	ACT order	HB3	Ups	Loc
1	*PFL0030c*	E	STt	1	*IT4var4*	E		1	*HB3var2csaA*	E	STt
2	*PFE1640w-ps*	A2	STt	2	*FCR3 var1csa*	A2	ST	2	*HB3var2csaB*	E	STt
3	*PFA0015c*	A1	STt	3	*IT4var3*	A1		3	*HB3var1csa*	A2	STt
4	*PFF0020c*	A1	STt	4	*R29var1*	A1	STt	4	*HB3var1*	A1	STt
5	*PFI1820w*	A1	STt	5	*IT4var18*	A1		5	*HB3var2*	A1	STt
6	*PF08_0141*	A1	STt	6	*IT4var22*	A1		6	*HB3var4*	A1	STt
7	*MAL8P1.207*	A1	STt	7	*IT4var2*	A1		7	*HB3var5*	A2	STt
8	*PF11_0008*	A1	STt	8	*IT4var60*	A1		8	*HB3var3*	A1	STt
9	*PFD0020c*	A1	STt	9	*IT4var64*	A1		9	*HB3var6*	A1	Cen
10	*PF13_0003*	A1	STt	10	*IT4var7*	A1		10	*HB3var30*	C1	Cen
11	*PF11_0521*	A1	STt	11	*IT4var8*	A1		11	*HB3var31*	C1	Cen
12	*PFD1235w*	A1	STt	12	*IT4var1*	C1	Cen	12	*Hb3var33*	C1	Cen
13	*PFD0615c*	C1	Cen	13	*IT4var5*	C1		13	*HB3var28*	C1	Cen
14	*PFD0625c*	C1	Cen	14	*IT4var28*	C2		14	*HB3var29*	C1	Cen
15	*PFD0630c*	C1	Cen	15	*IT4var47*	C1		15	*HB3var32*	C1	Cen
16	*PFD0995c*	C1	Cen	16	*IT4var16*	B2		16	*HB3var34*	C1	Cen
17	*PFD1000c*	C1	Cen	17	*IT4var27*	B3	Cen	17	*HB3var36*	C2	Cen
18	*PFD1015c*	C1	Cen	18	*FCR3 var3*	B2	Cen	18	*HB3var35*	C1	Cen
19	*PFF0845c*	C1	Cen	19	*FCR3 var2*	B1	Cen	19	*HB3var27*	B3	Cen
20	*PF07_0048*	C1	Cen	20	*IT4var11*	B1		20	*HB3var23*	B4	Cen
21	*PF07_0049*	C1	Cen	21	*IT4var13*	B1		21	*HB3var26*	B3	Cen
22	*PF07_0051*	C1	Cen	22	*IT4var17*	B1		22	*HB3var24*	B3	Cen
23	*MAL7P1.56*	C2	Cen	23	*IT4var19*	B1		23	*HB3var25*	B3	Cen
24	*PF08_0107*	C1	Cen	24	*IT4var25*	B1		24	*HB3var22*	B1	Cen
25	*PFL1960w*	C1	Cen	25	*IT4var26*	B1		25	*HB3var21*	B1	Cen
26	*PF07_0050*	B3	Cen	26	*IT4var29*	B1		26	*HB3var20*	B1	STc
27	*PFD0635c*	B3	Cen	27	*IT4var33*	B1		27	*HB3var17*	B1	Cen
28	*PFL1950w*	B4	Cen	28	*IT4var41*	B1		28	*HB3var19*	B1	Cen
29	*PF08_0103*	B3	Cen	29	*IT4var54*	B1		29	*HB3var18*	B1	Tc
30	*PFD1005c*	B1	Cen	30	*ITGvar*	B1		30	*HB3var12*	B1	Tc
31	*MAL7P1.50*	B1	Cen	31	*A4var tres*	B1	T	31	*HB3var13*	B1	Tc
32	*MAL7P1.55*	B1	Cen	32	*A4var ICAM*	B1	Tc	32	*HB3var14*	B1	Tc
33	*PF08_0106*	B1	Cen	33	*CS2var*	U	T	33	*HB3var8*	B1	Tc
34	*PFL1955w*	B1	Cen					34	*HB3var9*	B1	Tc
35	*PFL0935c*	B1	Cen					35	*HB3var15-inc*	B1	Tc
36	*PFF0010w*	B2	STc					36	*HB3var11*	B1	Tc
37	*PF08_0140*	B1	STc					37	*HB3var10*	B1	Tc

38	*PFF1580c*	B1	STc
39	*PFL0020w*	B1	STc
40	*PFA0005w*	B1	Tc
41	*PFA0765c*	B1	Tc
42	*PFB0010w*	B1	Tc
43	*PFB1055c*	B1	Tc
44	*PFC0005w*	B1	Tc
45	*PFC1120c*	B1	Tc
46	*PFD0005w*	B1	Tc
47	*PFD1245c*	B1	Tc
48	*PFE0005w*	B1	Tc
49	*PFF1595c*	B1	Tc
50	*MAL7P1.212*	B1	Tc
51	*MAL7P1.187*	B1	Tc
52	*PF08_0142*	B1	Tc
53	*MAL8P1.220*	B1	Tc
54	*PFI0005w*	B1	Tc
55	*PFI1830c*	B1	Tc
56	*PF10_0001*	B1	Tc
57	*PF10_0406*	B1	Tc
58	*PF11_0007*	B1	Tc
59	*PFL0005w*	B1	Tc
60	*PFL2665c*	B1	Tc
61	*MAL13P1.1*	B1	Tc
62	*MAL13P1.356*	B1	Tc

38	*HB3var7*	B1	Tc
39	*HB3var16*	B1	Tc

References

1. Duffy MF, Brown GV, Basuki W, Krejany EO, Noviyanti R, Cowman AF, Reeder JC: **Transcription of multiple var genes by individual, trophozoite-stage Plasmodium falciparum cells expressing a chondroitin sulphate A binding phenotype.** *Mol Microbiol* 2002, **43:** 1285-93.

2. Fernandez V, Chen Q, Sundstrom A, Scherf A, Hagblom P, Wahlgren M: **Mosaic-like transcription of var genes in single Plasmodium falciparum parasites.** *Mol Biochem Parasitol* 2002, **121:** 195-203.

3. Rowe JA, Moulds JM, Newbold CI, Miller LH: **P. falciparum rosetting mediated by a parasite-variant erythrocyte membrane protein and complement-receptor 1.** *Nature* 1997, **388:** 292-5.

4. Kraemer SM, Gupta L, Smith JD: **New tools to identify var sequence tags and clone full-length genes using type-specific primers to Duffy binding-like domains.** *Mol Biochem Parasitol* 2003, **129:** 91-102.
