# Supplementary material for: Patterns of gene recombination shape var gene repertoires in Plasmodium falciparum: comparisons of geographically diverse isolates
Source: BMC Genomics. 2007 Feb 7;8:45. doi: 10.1186/1471-2164-8-45 (PMC1805758; doi:10.1186/1471-2164-8-45)
Supplement: Additional file 1 — Schematic representation of 3D7 var genes. Genes are organized as in Figures 1 and 2. [file 1471-2164-8-45-S1.pdf]

3D7 gene name	Alias(es)	Ups	Extracellular Domain Structure (Predicted)										ATS	Dom Struc Type	Chr Loc	Var Group
PFA0015c	Type 3 var	A1	DBL1α1	DBL2ε									exon2A1	3	1L-STt	A1ST
PFF0020c	Type 3 var	A1	DBL1α1	DBL2ε									exon2A1	3	6L-SSTt	A1SST
PFI1820w	Type 3 var	A1	DBL1α1	DBL2ε									exon2A1	3	9R-STt	A1ST
PF11_0008		A1	DBL1α1	CIDR1γ	DBL2γ	DBL3δ	CIDR2β	DBL4β	C2				exon2A1	10a	11L-STt	A1ST
PF08_0141		A1	DBL1α1	CIDR1γ	DBL2β	C2	DBL3γ	DBL4β	DBL5ε				exon2A1	2c	8L-STt	A1ST
PF13_0003		A1	DBL1α1	CIDR1γ	DBL2β	C2	DBL3γ	DBL4δ	CIDR2β	DBL5β	C2		exon2A1	7	13L-STt	A1ST
PFD0020c		A1	DBL1α1	CIDR1α1	DBL2β	C2	DBL3γ	DBL4γ	DBL5δ	CIDR2β			exon2A1	8	4L-STt	A1ST
PF11_0521		A1	DBL1α1	CIDR1α1	DBL2β	C2	DBL3β	C2	DBL4δ	CIDR2γ			exon2A1	9	11R-Tt	A1T
MAL8P1.207	MAL7P1.1	A1	DBL1α1	CIDR1α1	DBL2β	C2	DBL3β	C2	DBL4γ	DBL5δ	CIDR2β		exon2A1	4	8R-STt	A1ST
PFD1235w		A1	DBL1α1	CIDR1α1	DBL2β	C2	DBL3β	C2	DBL4γ	DBL5δ	CIDR2β		exon2A1	4	4R-STt	A1ST
PFE1640w-ps	var1csa	A2	DBL1α1	CIDR1α1	DBL2β	C2	DBL3γ	DBL4ε	DBL5γ	DBL6β	DBL7ε			17	5R-Tt	A2T
PF08_0140		B1	DBL1α	CIDR1α1	DBL2β	C2	DBL3γ	DBL4δ	CIDR2β				exon2B	11a	8L-SSTc	B1SST
PFF1580c	MAL6P1.4	B1	DBL1α	CIDR1α	DBL2β	C2	DBL3γ	DBL4δ	CIDR2β	DBL5ε	DBL6ε	DBL7ε	exon2B	6	6R-STc	B1ST
PFL0020w		B1	DBL1α	CIDR1α	DBL2β	C2	DBL3γ	DBL4β	DBL5ε				exon2B	2a	12L-STc	B1ST
PFF0010w	MAL6P1.316	B2	DBL1α	CIDR1γ	DBL2β	C2	DBL3γ	DBL4β	DBL5ε				exon2B	2b	6L-STc	B2ST
PFB0010w		B1	DBL1α	CIDR1α	DBL2γ								exon2B	16	2L-Tc	B1T
PFA0005w		B1	DBL1α	CIDR1α	DBL2δ	CIDR2β							exon2B	1a	1L-Tc	B1T
PFA0765c		B1	DBL1α	CIDR1α	DBL2δ	CIDR2β							exon2B	1a	1R-Tc	B1T
PFB1055c		B1	DBL1α	CIDR1α	DBL2δ	CIDR2β							exon2B	1a	2R-Tc	B1T
PFC0005w		B1	DBL1α	CIDR1α	DBL2δ	CIDR2β							exon2B	1a	3L-Tc	B1T
PFC1120c		B1	DBL1α	CIDR1α	DBL2δ	CIDR2β							exon2B	1a	3R-Tc	B1T
PFD1245c		B1	DBL1α	CIDR1α	DBL2δ	CIDR2β							exon2B	1a	4R-Tc	B1T
PFE0005w		B1	DBL1α	CIDR1α	DBL2δ	CIDR2β							exon2B	1a	5L-Tc	B1T
PFF1595c	MAL6P1.1	B1	DBL1α	CIDR1α	DBL2δ	CIDR2β							exon2B	1a	6R-Tc	B1T
MAL7P1.212		B1	DBL1α	CIDR1α	DBL2δ	CIDR2β							exon2B	1a	7L-Tc	B1T
PF08_0142		B1	DBL1α	CIDR1α	DBL2δ	CIDR2β							exon2B	1a	8L-Tc	B1T
MAL8P1.220		B1	DBL1α	CIDR1α	DBL2δ	CIDR2β							exon2B	1a	8R-Tc	B1T
PF10_0406		B1	DBL1α	CIDR1α	DBL2δ	CIDR2β							exon2B	1a	10R-Tc	B1T
PF11_0007		B1	DBL1α	CIDR1α	DBL2δ	CIDR2β							exon2B	1a	11L-Tc	B1T
PFL0005w		B1	DBL1α	CIDR1α	DBL2δ	CIDR2β							exon2B	1a	12L-Tc	B1T
PFL2665c		B1	DBL1α	CIDR1α	DBL2δ	CIDR2β							exon2B	1a	12R-Tc	B1T
MAL13P1.1		B1	DBL1α	CIDR1α	DBL2δ	CIDR2β							exon2B	1a	13L-Tc	B1T
MAL13P1.356		B1	DBL1α	CIDR1α	DBL2δ	CIDR2β							exon2B	1a	13R-Tc	B1T
PFI0005w		B1	DBL1α	CIDR1α	DBL2δ	CIDR2γ							exon2B	1b	9L-Tc	B1T
PFI1830c		B1	DBL1α	CIDR1α	DBL2δ	CIDR2γ							exon2B	1b	9R-Tc	B1T
PF10_0001		B1	DBL1α	CIDR1α	DBL2δ	CIDR2γ							exon2B	1b	10L-Tc	B1T
MAL7P1.187	PF07_0139	B1	DBL1α	CIDR1α	DBL2δ	CIDR2β	DBL3ε						exon2B	14	7R-Tc	B1T
PFD0005w		B1	DBL1α	CIDR1α	DBL2γ	DBL3δ	CIDR2β						exon2B	12	4L-Tc	B1T
PF08_0106		B1	DBL1α	CIDR1α	DBL2δ	CIDR2β							exon2B	1a	8-C	B1C
PFL1955w		B1	DBL1α	CIDR1α	DBL2δ	CIDR2β							exon2B	1a	12-C	B1C
PFD1005c		B1	DBL1α	CIDR1α	DBL2δ	CIDR2γ							exon2B	1b	4-C	B1C
MAL7P1.50		B1	DBL1α	CIDR1α	DBL2δ	CIDR2γ							exon2B	1b	7-C	B1C
MAL7P1.55		B1	DBL1α	CIDR1α	DBL2δ	CIDR2γ							exon2B	1b	7-C	B1C
PFL0935c		B1	DBL1α	CIDR1α	DBL2δ	CIDR2γ							exon2B	1b	12-C	B1C
PF08_0103		B3	DBL1α	CIDR1α	DBL2δ	CIDR2β							exon2B	1a	8-C	B3C
PFD0635c		B3	DBL1α	CIDR1α	DBL2δ	CIDR2γ							exon2B	1b	4-C	B3C
PF07_0050		B3	DBL1α	CIDR1α	DBL2β	C2	DBL3γ						exon2B	15	7-C	B3C
PFL1950w		B4	DBL1α	CIDR1α	DBL2β	C2	DBL3δ	CIDR2β					exon2B	5	12-C	B4C
PFD0615c		C1	DBL1α	CIDR1α	DBL2δ	CIDR2β							exon2B	1a	4-C	C1C
PFD0625c		C1	DBL1α	CIDR1α	DBL2δ	CIDR2β							exon2B	1a	4-C	C1C
PFD1015c		C1	DBL1α	CIDR1α	DBL2δ	CIDR2β							exon2B	1a	4-C	C1C
PF07_0048		C1	DBL1α	CIDR1α	DBL2δ	CIDR2β							exon2B	1a	7-C	C1C
PFL1960w		C1	DBL1α	CIDR1α	DBL2δ	CIDR2β							exon2B	1a	12-C	C1C
PF07_0051		C1	DBL1α	CIDR1α	DBL2δ	CIDR2β							exon2A2	1a	7-C	C1C
PFD0630c		C1	DBL1α	CIDR1α	DBL2δ	CIDR2γ							exon2B	1b	4-C	C1C
PFD1000c		C1	DBL1α	CIDR1α	DBL2δ	CIDR2γ							exon2B	1b	4-C	C1C
PF07_0049		C1	DBL1α	CIDR1α	DBL2δ	CIDR2γ							exon2B	1b	7-C	C1C
PF08_0107		C1	DBL1α	CIDR1α	DBL2δ	CIDR2γ							exon2B	1b	8-C	C1C
PFD0995c		C1	DBL1α	CIDR1α	DBL2δ	CIDR2γ							exon2A2	1b	4-C	C1C
PFF0845c	MAL6P1.252	C1	DBL1α	CIDR1α	DBL2β	C2	DBL3δ	CIDR2β					exon2A2	5	6-C	C1C
MAL7P1.56		C2	DBL1α	CIDR1α	DBL2δ	CIDR2β							exon2B	1a	7-C	C2C
PFL0030c	var2csa	E	DBL1	DBL2	DBL3	DBL4ε	DBL5ε	DBL6ε					exon2E	13	12L-SSTt	ESST
