# Supplementary figures and images for: Patterns of gene recombination shape var gene repertoires in Plasmodium falciparum: comparisons of geographically diverse isolates
Source: BMC Genomics. 2007 Feb 7;8:45. doi: 10.1186/1471-2164-8-45 (PMC1805758; doi:10.1186/1471-2164-8-45)

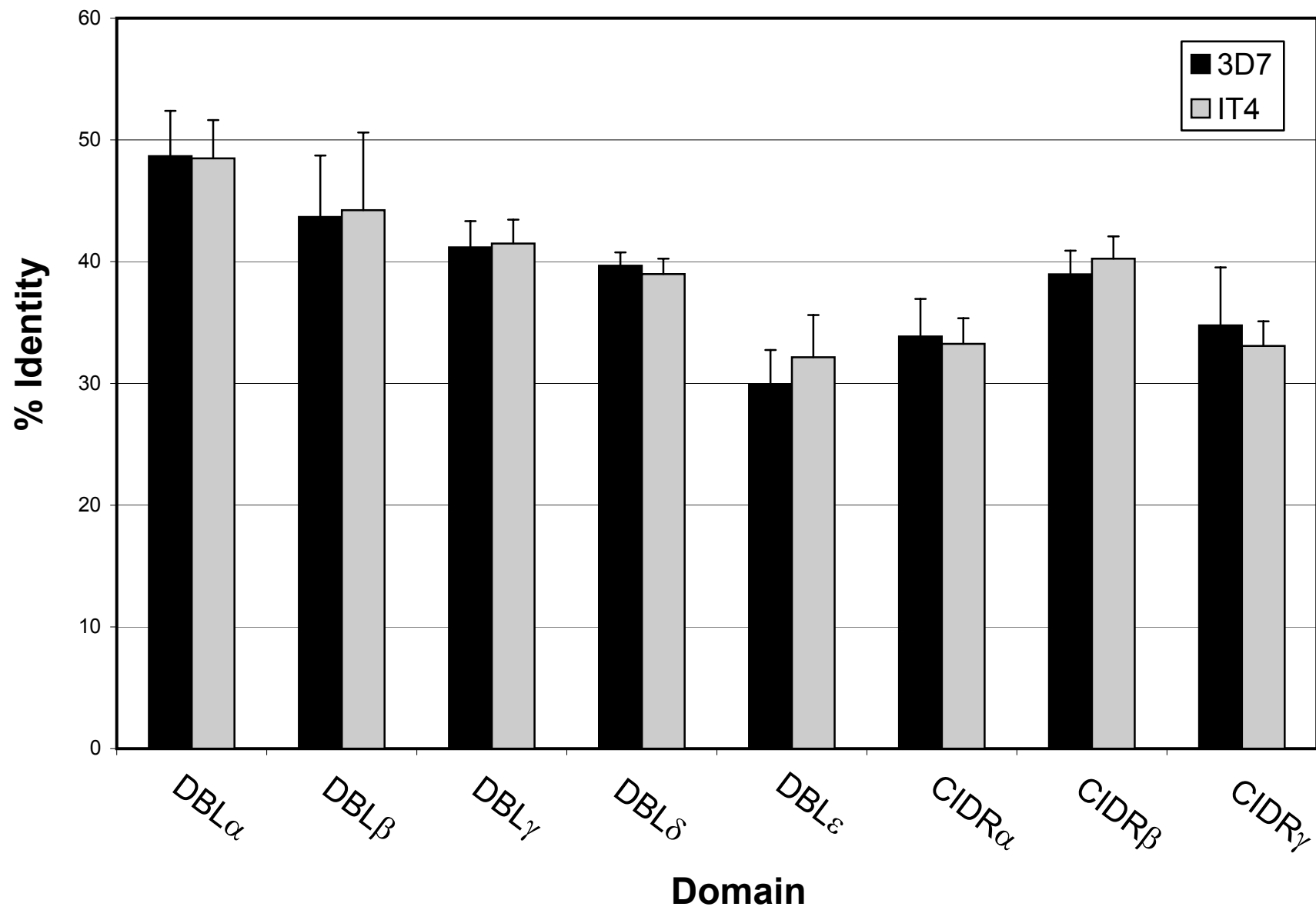

Supplement: Additional file 2 — Amino acid identity of PfEMP1 adhesion domains from 3D7 and IT4 parasite isolates. Pair-wise comparisons were performed for all of the adhesion domains in 3D7 and IT4 PfEMP1 proteins. For each individual domain, the average identity to all other IT4 or 3D7 domains of that type was determined. These means were then averaged for 3D7 and IT4. Error bars represent one standard deviation of the mean of means. [file 1471-2164-8-45-S2.pdf]
