# Supplementary material for: Patterns of gene recombination shape var gene repertoires in Plasmodium falciparum: comparisons of geographically diverse isolates
Source: BMC Genomics. 2007 Feb 7;8:45. doi: 10.1186/1471-2164-8-45 (PMC1805758; doi:10.1186/1471-2164-8-45)
Supplement: Additional file 3 — Amino acid identity between PfEMP1 proteins of different Ups types. Alignment "hits" collected from the dot plot shown in Figure 7 were plotted per amino acid position of individual proteins. Graphs represent 3D7 PfEMP1 protein alignment "hits" (including the var1csa pseudo-gene) plotted against individual IT4 or 3D7 PfEMP1 proteins. Based upon the distribution of var genes in the 3D7 isolate, the maximum number of hits at individual amino acid positions for genes of each promoter type is UpsA (9), UpsB (22), UpsC (13), UpsA2 (formerly UpsD; 1), UpsE (1), and Ups B2-4 (13) [6,11]. Previously defined DBL homology blocks B, D and H are labeled [10]. [file 1471-2164-8-45-S3.pdf]

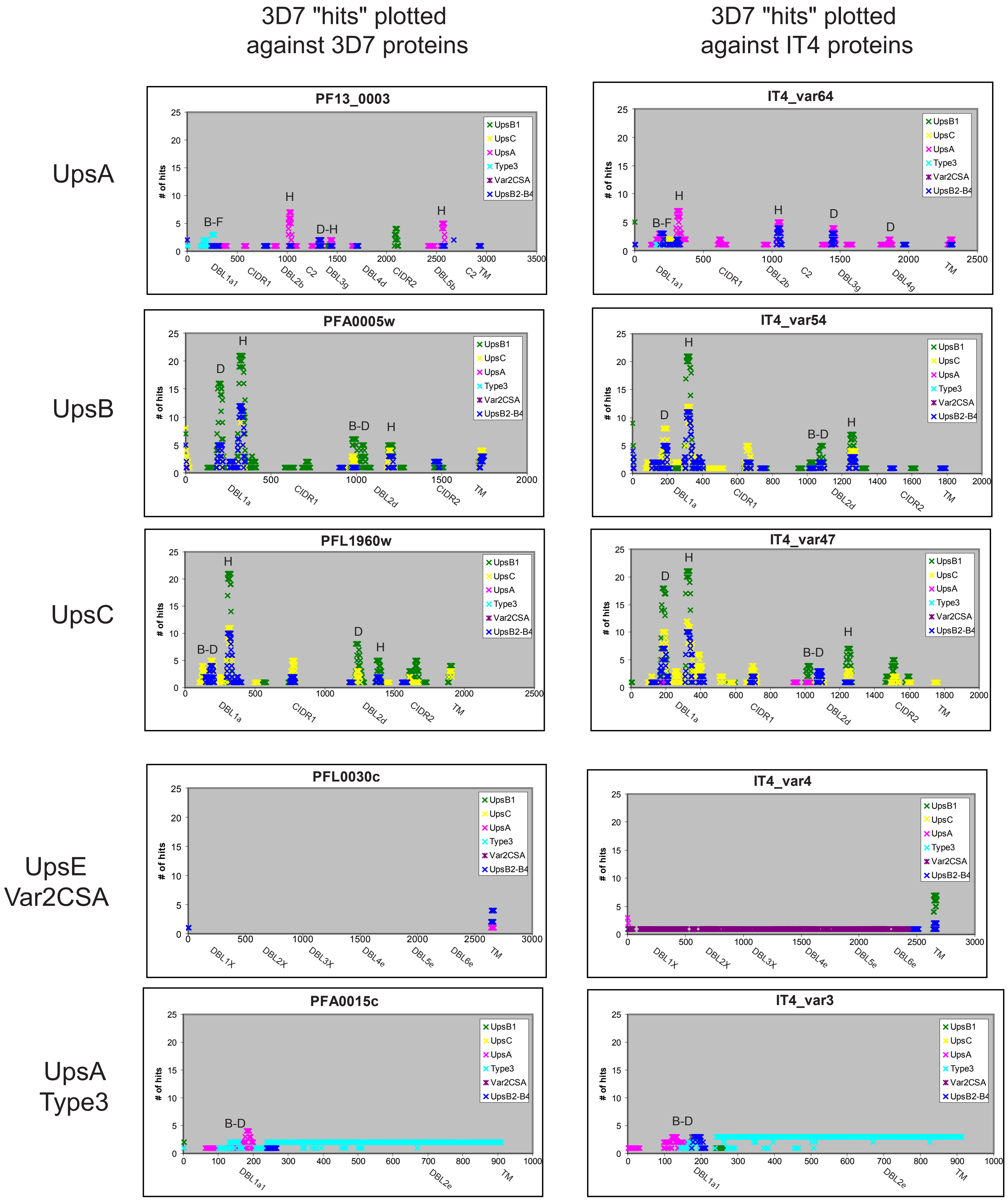
3D7 "hits" plotted against 3D7 proteins
3D7 "hits" plotted against IT4 proteins
UpsA
UpsB
UpsC
UpsE Var2CSA
UpsA Type3
PF13_0003
IT4_var64
PFA0005w
IT4_var54
PFL1960w
IT4_var47
PFL0030c
IT4_var4
PFA0015c
IT4_var3
# of hits
UpsB1
UpsC
UpsA
Type3
Var2CSA
UpsB2-B4
B-F
H
D-H
D
B-D
DBL1a1
CIDR1
DBL2b
C2
DBL3g
DBL4d
CIDR2
DBL5b
TM
DBL4g
DBL1a
DBL2d
DBL1X
DBL2X
DBL3X
DBL4e
DBL5e
DBL6e
DBL2e
